# Framing Inequality as Advantage versus Disadvantage: A Systematic Review of Effects and a Two-Step Model to Explain Them

**DOI:** 10.1177/10888683251333458

**Published:** 2025-04-30

**Authors:** Annette Malapally, Nicole Methner, Maike Braun, Sophia Wittenborn, Susanne Bruckmüller

**Affiliations:** 1 Friedrich-Alexander-Universität Erlangen-Nürnberg, Erlangen, Germany; 2 University of Hohenheim, Stuttgart, Baden-Württemberg, Germany

**Keywords:** equivalency framing, inequality, advantage, privilege, disadvantage, discrimination, conceptual integrative framework

## Abstract

**Academic Abstract:**

Although disadvantage and advantage jointly make up inequality, inequality is often one-sidedly framed as disadvantage. Concurrently, efforts to raise awareness for advantages are growing. Many studies have examined whether and how it matters if inequality is framed as advantage or disadvantage. However, empirical and conceptual integration of this work is lacking. For empirical integration, we systematically reviewed 71 experimental studies in 36 documents (*n* = 20,063). These investigated many different variables, but often only once, or with inconsistent findings. Framing manipulations varied in ways that could bias effects. Summarizing consistent effects, we conclude that framing can influence how people perceive and react to inequality, but this is contingent on moderators. For conceptual integration, we developed a two-step model, which defines (dis)advantage frames and aims to explain why (Step 1) and how (Step 2) they influence which variables, to help this exciting research field move forward in a more systematic way.

**Public Abstract:**

Inequality is one of the biggest challenges of our time. Both disadvantage and advantage are mechanisms that create and maintain inequality. However, there is often a one-sided focus on disadvantage, though awareness for advantage is growing slowly. This makes it important to ask whether and to what extent it matters if inequality is understood and talked about in terms of disadvantages or advantages. We analyzed and summarized previous studies that investigated these questions and developed an integrating conceptual model. Taken together, the way we talk about inequality can influence how people perceive and react to it, for example, how (il)legitimate they find it and what they want to do about it. Neither talking about inequality as advantage nor as disadvantage is per se more conducive to challenging or maintaining inequality. A balanced understanding of inequality seems necessary to fully understand the issue and to develop effective interventions.

## Introduction

Reducing inequalities is one of the 17 global sustainable development goals of the United Nations who have called inequality one of the most pressing issues of our time (United Nations, 2022). While social inequality refers to an asymmetry in resources and opportunities between social groups, there is also asymmetry in how we think, talk about, and research inequality (Phillips et al., 2022). Although disadvantage and advantage jointly make up systems of inequality, participants in experiments (Malapally & Bruckmüller, 2024; Phillips & Jun, 2022), journalists (for gender and racial inequality; Jun et al., 2022), and lay people talking about inequality in social media (Malapally et al., 2024; Malapally & Bruckmüller, 2024) speak more about disadvantage than about advantage, and scholars research disadvantaging more than advantaging mechanisms (Phillips et al., 2022).

While this is an inequality in and of itself, a one-sided perspective on disadvantage also limits the range of possible explanations and solutions in discussions of inequality (Bruckmüller & Braun, 2020; Nixon, 2019). Critical scholars have long argued that focusing on disadvantage facilitates the invisibility of privilege and favoritism (e.g., McIntosh, 1988; Pratto & Stewart, 2012)—which helps to uphold inequality (Brewer, 1999; DiTomaso, 2015). In response, awareness exercises and trainings try to broaden understandings of inequality by confronting people with their privileges (e.g., Ehrke et al., 2020; Pickering, 2023). Such efforts and the pervasive one-sidedness in discourse about the key societal issue of inequality render the questions important, how problematic a one-sided focus on disadvantage really is, and which (un)desirable effects it may have to turn the lens and make advantages more visible. In short, whether and to what extent does it matter if inequality is understood and talked about in terms of advantages or disadvantages?

There are a considerable number of studies on this question, and research interest has risen in recent years (see below). This has created a relatively young and dynamic, but also hard to oversee, research field studying effects of framing inequality as advantage versus disadvantage. These studies employ a wide range of methods in diverse inequality domains and study effects on many different dependent variables and moderators. Critically, there is no unified theorizing or conceptual framework, which would predict when, why, and how (dis)advantage framing effects occur. It is, thus, a necessary next step to systematically summarize the scattered findings on an empirical level and to integrate them on a conceptual level. Thus, we aim to do both: give a systematic and comprehensive overview of research on (dis)advantage framing and outline a conceptual framework to integrate previous research and identify directions for future work.

## Scattered and Inconsistent Approaches and Findings in (Dis)Advantage Framing Research

### What Is (Dis)Advantage Framing?

Variations in how people speak and write about issues are referred to as differences in *framing* (Druckman, 2011; Entman, 1993). In the context of inequality, *disadvantage framing* focuses on disadvantages of certain social groups, while *advantage framing* focuses on advantages of other groups (e.g., Phillips et al., 2022). For example, economic inequality can be framed as the poor having less than the rich (disadvantage frame), or as the rich having more than the poor (advantage frame). These frames are a form of *equivalency framing*. They present logically equivalent information in semantically or syntactically different ways, i.e., they only vary *how* identical information is presented (Scheufele & Iyengar, 2014; Tversky, 1977). This differentiates equivalency framing from *emphasis* framing, which varies *what* is communicated (Cacciatore et al., 2016; Druckman, 2011). Examples for different emphasis frames of inequality are thematic (e.g., national trends) versus episodic frames (e.g., the situation of one family, see Iyengar, 1990).

Advantage versus disadvantage framing, the equivalency framing we are specifically interested in here, is a form of *comparative framing*, that is, variations in who is the target and who is the referent in a comparison (e.g., whether A [target] is compared to B [referent] or B to A, Tversky, 1977; Tversky & Gati, 1978). This differentiates comparative framing from other forms of equivalency framing, which use different wordings to describe identical information, such as Tversky and Kahneman’s (1981) classic work on gain and loss framing (e.g., lives lost vs. lives saved). In this article, we use the term *(dis)advantage framing* to refer to equivalency framing of inequality as advantage versus disadvantage. We focus on this type of framing here because varying *only* the comparative focus (and not other features of a message) allows investigating *only* the effects of focusing on disadvantage versus advantage (and not the effects of specific wording or content).

### How Have (Dis)Advantage Framing Effects Been Investigated?

In recent years, a fast-growing number of studies tested effects of (dis)advantage framing, revealing that it can affect how people perceive, explain, and react to inequality (e.g., Bruckmüller & Braun, 2020; Dietze & Craig, 2021; Dover, 2022; Phillips et al., 2022). While most of this research experimentally varied framing by asking participants to read equivalent texts with either advantage or disadvantage framing, the respective studies differ in many other ways. The underlying theoretical approaches seem to vary widely, that is, between prospect theory (e.g., Ash & Schmierbach, 2013), norm theory (e.g., Bruckmüller et al., 2017a), social identity theory (e.g., Powell et al., 2005), and theoretical models specifically developed for the context of inequality framing (e.g., Chow et al., 2010). In addition, previous research has investigated (dis)advantage framing in different domains of inequality (e.g., gender, racial, or class inequality). It has examined effects on a wide range of dependent variables (e.g., ingroup identification, perceptions of inequality, affect, group evaluations, and support for interventions), and it has considered diverse moderators and mediators. Across studies, identical variables, such as the perceived legitimacy of inequality, were sometimes considered dependent variables (e.g., Bruckmüller et al., 2017a) and sometimes moderators (e.g., Harth et al., 2008).

Moreover, framing effects have been investigated with diverse measurement approaches – most often, items on Likert scales (e.g., Lowery et al., 2012), but also including physiological reactions (Dover, 2022), communication behavior in open responses (e.g., Bruckmüller & Braun, 2020), and other behavior (e.g., user engagement on Facebook; Dietze & Craig, 2021). Yet, many of these variables seem to have been examined only with regard to one particular kind of inequality (e.g., gender or racial inequality), or even only in one study or set of studies reported in one manuscript (see below). Moreover, some of the reported effects seem to be contradictory. This includes individual effects on specific dependent variables (see below), but also the more overarching conclusions that this research provides, for example, with regard to which framing is more conducive to challenging inequality.

### Which Framing Is More Conducive to Challenging versus Maintaining Inequality?

At first glance, it may seem like disadvantage framing is more conducive to challenging inequality, and advantage framing more to maintaining it. For example, a focus on disadvantage can make discrimination easier to recognize (Phillips et al., 2022), increase sympathy for the disadvantaged (Harth et al., 2008), make inequality seem less legitimate (Bruckmüller et al., 2017a), and increase willingness to act against inequality (Dietze & Craig, 2021)—which means that advantage framing makes discrimination *harder* to recognize, *decreases* sympathy, makes inequality *more* legitimate, and *decreases* willingness to act. Further, a focus on advantage can also be threatening to members of privileged groups (Dover, 2022, Lowery et al., 2007) and lead to reactance, for example, in the form of denying privilege (Phillips & Lowery, 2015).

However, there are also findings that suggest the opposite, that is, that advantage framing is more conducive to challenging inequality and disadvantage framing to maintaining it. For example, advantage framing can lead advantaged people to express less racism (Powell et al., 2005), evaluate disadvantaged groups less negatively (Rosette & Koval, 2018), and support redistributive policies more (Lowery et al., 2012)—which means that disadvantage framing leads to *more* expression of racism, *more* negative evaluations, and *less* support for redistributive policies.

In sum, (dis)advantage framing research is an emerging field investigating diverse framing effects, resulting in a puzzle of findings calling for systematic empirical integration. Inconsistencies in the conceptual status of variables, and even more so the plethora of theoretical approaches dis(advantage) framing research draws on, highlight the need for conceptual integration.

### The Present Research (Questions)

Necessary next steps for the growing, quite heterogeneous field of (dis)advantage framing research are to systematically synthesize this research on an empirical and conceptual level. This is important to advance our understanding of why and how (dis)advantage framing can affect perceptions of and reactions to inequality. It is also important for inequality research more generally, which is a central topic in the social sciences. Yet, this research tends to (one-sidedly) address inequality from a perspective of disadvantage (Phillips et al., 2022), which may have critical consequences, such as limiting our understanding of the roots and consequences of inequality (Brewer, 1999; DiTomaso, 2015). It may also reproduce inequalities, for example, due to blindness for advantaging mechanisms, such as permissiveness, helping, nepotism, or structural advantages (Phillips et al., 2022). Thus, the present research first aims to provide the necessary empirical integration, by ways of a systematic literature review. This review identifies the methodological scope of earlier studies, underlying theory, and the effects of (dis)advantage framing, including (hidden) moderators and mediators. Second, building on these insights, we aim to provide the necessary conceptual integration of this research field, by developing an integrative conceptual framework of (dis)advantage framing research.

#### Systematic Literature Review of (Dis)Advantage Framing Effects

To provide empirical integration, we conducted a systematic review with the aim to capture the many different dependent variables, moderators, populations, and inequality domains investigated in earlier studies. We included studies that experimentally manipulated advantage versus disadvantage framing of any kind of inequality between social groups and examined its effects on any outcome. We only included experiments because we were interested in the *causal* effects of (dis)advantage framing. Our research questions (RQ) were:

*RQ1.* What is the (methodological) scope of previous studies on (dis)advantage framing? Are there any research gaps in (a) which study designs have been used and how framing was manipulated, and in (b) which perspectives (e.g., of disadvantaged vs. advantaged groups), (c) which domains of inequality (e.g., race, gender, class), and (d) which dependent variables, moderators, and mediators have been investigated and how they were measured?
*RQ2.* How does (dis)advantage framing (a) affect recipients’ understanding of, and their reactions to, inequality? And (b) can (hidden) moderators be identified?
*RQ3.* Which theories were used to explain (dis)advantage framing effects?

#### An Integrative Conceptual Framework of (Dis)Advantage Framing Effects

To provide conceptual integration, we developed a two-step model of (dis)advantage framing effects. This model extends earlier theorizing (Chow et al., 2010; Phillips et al., 2022) in several ways. First, it provides an explicit definition of (dis)advantage framing. This will help researchers to construct theory-driven framing manipulations, which avoid confounding with other types of framing (e.g., emphasis framing). Second, by identifying a common denominator in previous theoretical approaches, the model proposes an explicit rationale *why* (dis)advantage framing can affect recipients’ responses to inequality. Third, to help deduct predictions for *how* (dis)advantage framing affects responses, the model also provides a classification of dependent variables and potential moderators. This systemizes previous findings, thereby facilitating the recognition of (previously overlooked) moderators and other research gaps. Fourth, the proposed model integrates previous theoretical approaches. It shows, on the one hand, why the previously applied plethora of theoretical approaches is necessary to make predictions for *how* the plethora of studied outcome variables is affected by (dis)advantage framing in different inequality domains and communication contexts. On the other hand, it shows how these different approaches can be integrated by identifying one underlying process for these varied effects (the *why*, see second point). Fifth, the two-step model allows us to derive testable, novel predictions for (dis)advantage framing effects, providing important directions for future research.

#### Positionality Statement

Inequality is intersectional. Thus, we, like most other people, have experiences of belonging to an advantaged group, for example, on the basis of being White, or our citizenship in a WEIRD country, and we also have experiences of belonging to a disadvantaged group, for example, on the basis of our gender, or, for some of us, our sexual orientation or social-class background. On the one hand, this may allow us to understand inequality from various perspectives. On the other hand, our experiences are also a source of bias because we are implicated in most of the inequalities that we write about as either someone who benefits or someone who suffers from them. For example, just like our participants, we are likely motivated to protect the moral image of our group when a privileged identity is salient, and to draw attention to our deprivation, when a disadvantaged identity is made salient. Further, our demographic backgrounds are quite similar, with all authors being White women from a WEIRD country, with a university education in psychology, and German as a first language. A more diverse background may have challenged our (implicit) understanding of language and inequality. To broaden the range of included studies in the systematic literature review across disciplines and conceptual approaches, we used a very broad set of keywords and screened a vast corpus of (mostly non-relevant) manuscripts.

## Systematic Literature Review

### Method

#### Transparency and Openness

In the methods and results sections, we adhere to the PRISMA 2020 guidelines for reporting systematic reviews (Page et al., 2021). We report the complete search strategy, all data inclusion and exclusion criteria, and all methods for data extraction and synthesis. All data for this project, along with relevant code, coding schemes, as well as supplementary tables and figures, are available on the OSF (osf.io/etq82). All analyses were run with R 4.3.0 (R Core Team, 2023) and the packages *irrCAC* (Gwet, 2019), *gmodels* (Warnes et al., 2022), *psych* (Revelle, 2021), and *jtools* (Long, 2020). The transparency of our data, analytic methods, and research materials meet PSPR’s TOP guidelines. This review project had no review protocol and was not preregistered because of its exploratory nature. One of our central goals was to describe the methodological scope of previous studies (including central variables), so we did not know beforehand which dependent variables, moderators, or mediators we would analyze, or which methods of data extraction and synthesis would be feasible. In the following sections, we will outline our methodology in broad strokes and refer to specific tables in the supplementary material for further details.

#### Search Strategy

The databases PsycInfo, Psychology and Behavioral Sciences Collection, Web of Science,^1^ Business Source Complete, Scopus, ERIC, Applied Social Sciences Index & Abstracts, and International Bibliography of the Social Sciences, as well as ResearchGate and the OSF preprint search engine were queried on February, 15, 2023. We had three groups of search terms connected by AND-operators, the first containing terms related to framing (e.g., “equivalen* fram*,” “descr* differences”), the second related to inequality (e.g., “inequality,” “discrimin*), and the third related to experiments (e.g., “experiment*,” “manipulat*”; for a full list, see Supplemental Table B1). This aimed to cover all experiments on the effects of (dis)advantage framing on any outcome.

**Figure 1.**
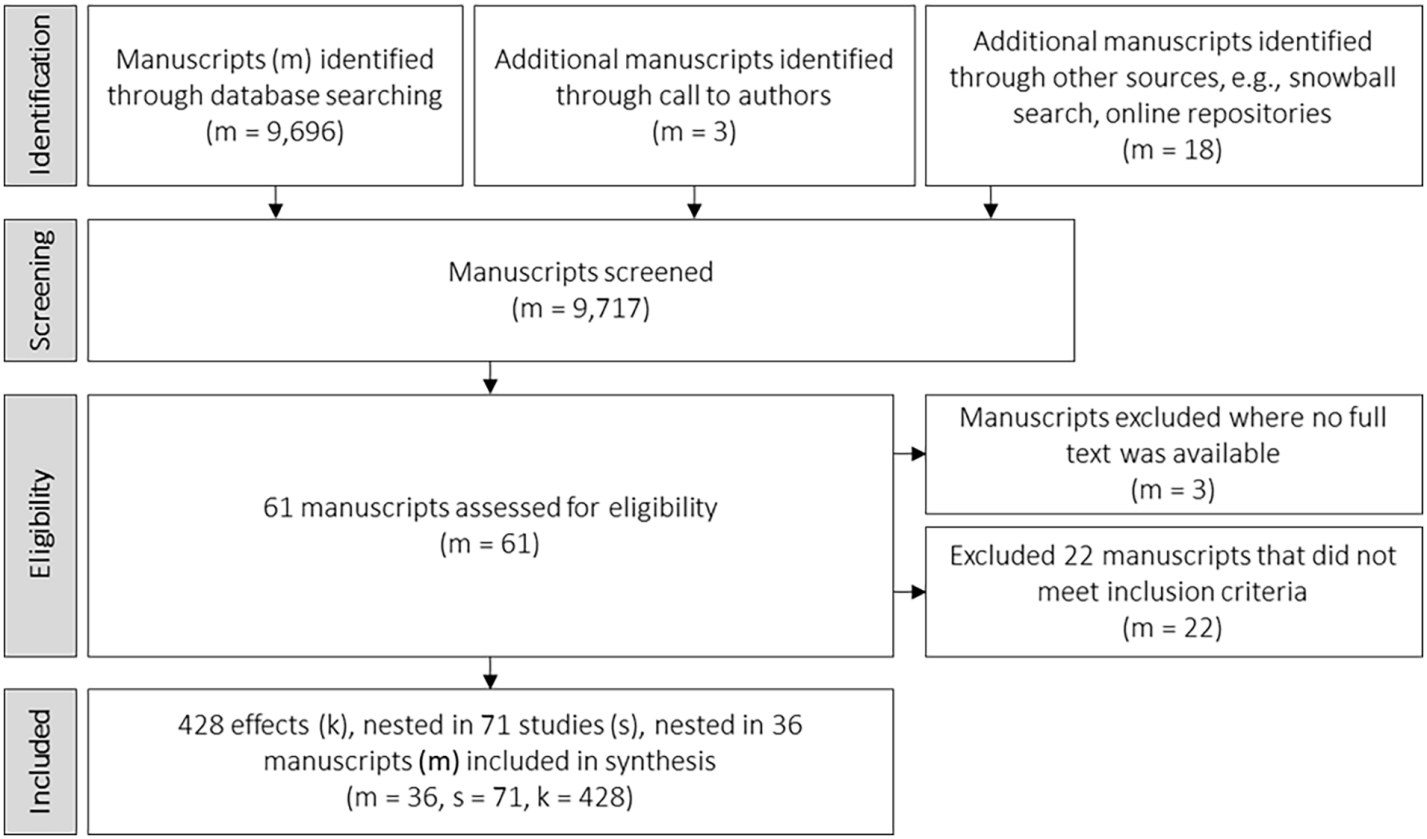
Data Collection Strategy and Number of Retrieved Manuscripts.

Additional manuscripts were retrieved through snowball search in earlier work and publication lists of authors of eligible manuscripts. Further, a call for (un)published data was sent to authors of eligible or closely related manuscripts and to mailing lists of scientific associations in the fields of psychology and communication science. Duplicate manuscripts were removed. Figure 1 shows the full data collection process and the number of retrieved manuscripts. In total, the systematic review is based on 36 documents, comprising 71 studies, and 428 unique effects, involving 20,063 participants in classical psychological experiments and 143,201 participants in two experiments run on Facebook (Dietze & Craig, 2021).

#### Appraisal of Eligibility

Of the 9,717 retrieved manuscripts, 100 were screened for eligibility by three raters on the basis of their abstract (Fleiss’ κ = .78; see Supplemental Table B2 for screening instructions). The three raters then separately screened the remaining manuscripts. The majority of retrieved manuscripts were not about (dis)advantage framing effects. Excluded manuscripts ranged from those on gain- versus loss framing of medical interventions, to image simplification in the context of pattern recognition, and business models in emerging markets.^2^

After screening, the included manuscripts were again rated for eligibility, on the basis of their full text. Three manuscripts were excluded because we could not retrieve a full text, not even by attempts to contact the original authors. The first author and a second independent rater coded 10 out of the 58 remaining manuscripts (Gwet’s AC1 = 1.00). The first author then coded the other 48 manuscripts. After considering the full text, 22 manuscripts were excluded because they did not meet inclusion criteria. Table 1 gives an overview of these criteria and examples of excluded studies (cf. Roberts et al., 2019).

**Table 1.** Inclusion and Exclusion Criteria.

	Inclusion criteria	Exclusion criteria	Example for excluded studies
Design	Experimental within- or between-subjects design	Articles without empirical data	Chow et al.’s (2010) work is a theoretical article on (dis)advantage framing.
Independent variables	Advantage vs. disadvantage framing was manipulated as an independent variable in at least two separate experimental conditions.		Iyer et al. (2003, Study 1) did not manipulate framing, but measured belief in White privilege vs. racial discrimination.
		Framing was only a dependent variable.	-
		The effect of advantage *or* disadvantage framing was compared against a control group.	Phillips and Lowery (2015) compared advantage framing to a control condition with no specific framing.
Dependent variables	Measurement of framing effects on any outcome		-
Framing operationalization	The manipulation was a form of equivalency framing, i.e., the presented inequality information was equivalent in content between the experimental conditions.	The manipulation was exclusively a form of emphasis or issue framing.	Hegarty et al. (2020) varied whether straight couples were compared to gay couples or vice versa, but also varied which group was advantaged and which was disadvantaged in the same experimental condition.
		The groups which were compared to each other were varied.	—
		The manipulation exclusively presented information about other issues (than inter-group inequality).	Howlett et al. (2021) presented inequality between individuals with no reference to their social group.
Participants	Any non-clinical population of adults over 18		—
Publication time	Published at any time		—
Outlets	(Non) peer-reviewed journals, conference papers, dissertations, Bachelor’s or Master’s theses, book chapters, government or company reports, or unpublished	If there were several manuscripts reporting the same data (e.g., dissertations and journal articles), any source but the one published first was excluded.	The data reported in Dover (2017) is also included in Dover (2022).
Languages	English, German, Spanish, French		—

#### Data Extraction

Out of the 36 eligible manuscripts, 10 were coded by two raters to establish reliability of the coding scheme. We coded which theories were used to explain framing effects. Then, to determine the (methodological) scope of studies, we coded characteristics of the samples, procedures, framing manipulations, dependent variables, and statistical effects (see Supplemental A for the coding scheme or Supplemental Table B3 for a summary; for reliability scores, see Supplemental Table B4). All coding categories showed substantial to perfect reliability (theoretical underpinnings: AC1 = .62, demographics: AC1 = .66, all other categories: .81 ≤ AC1 ≤ 1.00; Gwet, 2014; Landis & Koch, 1977). The first author then coded the remaining 26 manuscripts.

#### Data Synthesis

The following results and discussion section is structured along the research questions. First, we describe the methodological scope and research gaps of previous studies with regard to which study designs have been used (RQ1a) and which groups (RQ1b), domains (RQ1c), and variables (RQ1d) have been investigated. Dependent variables were clustered into categories through an iterative, data-driven process. Two independent raters created clusters of similar variables and reconciled them through discussion; this draft was then discussed by all authors and consolidated into the final clustering of eight categories (physiological reactions, perceptions and attributions of inequality, affect, attitudes, ingroup identification, behavior[al intentions], and other variables).

Second, we summarize previous findings on (dis)advantage framing effects (RQ2a) and their moderators (RQ2b). However, effect sizes could not be aggregated across dependent variables due to the heterogeneity of studies. Thus, in the main manuscript, we present a systematic overview, supported by graphical and tabular summaries. In the supplement, we provide detailed tables of all included effects (Supplemental Tables B6–B18). Finally, we describe the theories used to explain (dis)advantage framing effects and whether these theories were used to derive hypotheses (RQ3).

### Results and Discussion

#### The Methodological Scope of Previous Studies and Research Gaps (RQ1)

The methodological scope of previous studies is summarized in Table 2. We refer to the supplement for a detailed overview of all coding categories concerning methodology (Supplemental Table B5). Below, we will point out the most important conclusions regarding the design (RQ1a), investigated populations (RQ1b), contexts (RQ1c), and dependent variables (RQ1d).

**Table 2.** Methodological Scope of Previous Studies on (Dis)Advantage Framing.

Aspect	Details
Time of publication	2005–2023, with over half published since 2017
Place of investigation	59.2% U.S. 25.4% Germany 15.4% Others (Italy, Nepal, Norway, U.K.)
Study design	
Experimental design	All cross-sectional, between-subjects, at least two conditions (advantage vs. disadvantage framing)
Inequality Domains	40.9% racial inequality 23.9% class inequality 12.7% gender inequality 22.5% other inequalities (e.g., between countries, students of different subjects)
Manipulation of framing	
Legitimacy	35.2% inequality portrayed as illegitimate 45.1% ambiguous Two articles manipulated legitimacy (Harth, 2007; Salgado & Aguilar, 2022).
Experimenters	57.8% participants not aware of experimenters’ social group 11.3% members of the advantaged group 2.8% members of disadvantaged group 1.4% member of both groups 25.4% members of third group One article manipulated experimenters’ group membership (Greenaway et al., 2017).
Sample Sizes	Ranged from 29 to 2,899 participants (*Md* = 177) Additionally, one study conducted two experiments through an internal Facebook algorithm on 72,324 and 67,491 people, respectively (Dietze & Craig, 2021).

##### Experimental Design and Manipulations (RQ1a)

There is a growing body of research on (dis)advantage framing, with a strong focus on online studies conducted in the United States and Europe. All studies were cross-sectional and almost all measured framing effects in single, one-sided acts of communication (see Braun et al., 2023a, for an exception). Beyond that, methodologies varied considerably, for example, in the number of experimental factors and conditions, the use of control groups, and the source of information (e.g., disadvantaged, advantaged, or third group, no information reported). Manipulations also varied in whether they only varied the comparison focus to manipulate framing, or (also) used different terms (e.g., advantage versus privilege), in whether they described inequality as (il)legitimate, and in which kinds of outcomes (e.g., health, money) they described.

Importantly, these (possibly unintentional) variations in framing manipulations may account for discrepancies in findings between studies. For example, inequality was often (implicitly) portrayed as illegitimate, although legitimacy can moderate framing effects (e.g., Harth et al., 2008). The information source was sometimes a disadvantaged or an advantaged group member, although group membership is also a moderator (e.g., Greenaway et al., 2017). Terms such as “(dis)advantage,” “discrimination,” or “privilege” were used interchangeably, although they may have effects beyond the strict variation of comparative framing (cf. Quarles & Bozarth, 2022). In sum, study designs varied considerably and in ways which may have affected results.

##### Investigated Populations (RQ1b)

A study matrix was used to break down which variables were investigated for which participants and which inequality domain (see Table 3). Participants from advantaged groups were overrepresented, while disadvantaged groups were mostly included as participants in studies on class and gender inequality (e.g., Dietze & Craig, 2021, Bruckmüller & Braun, 2020), and third groups were mostly included in studies on class inequality (e.g., Bruckmüller et al., 2017a). Perceptions and attributions of inequality were well-researched. For example, perceived legitimacy was examined for racial (e.g., Lowery & Wout, 2010), class (e.g., Braun et al., 2023b), gender (e.g., Bruckmüller et al., 2012), and other inequalities (e.g., Greenaway et al., 2017). Other variables were mostly researched for one group in one domain (e.g., ingroup identification of advantaged groups for racial inequality; Chow et al., 2008). While the predominance of samples from advantaged groups creates a critical research gap per se, this is also important because group membership can moderate framing effects (Lowery & Wout, 2010). In sum, there was an imbalance in what was investigated for whom and in which context, making overall conclusions difficult.

**Table 3.** Study Space Analysis of Investigated Dependent Variables, Inequality Domains, and Participants Groups.

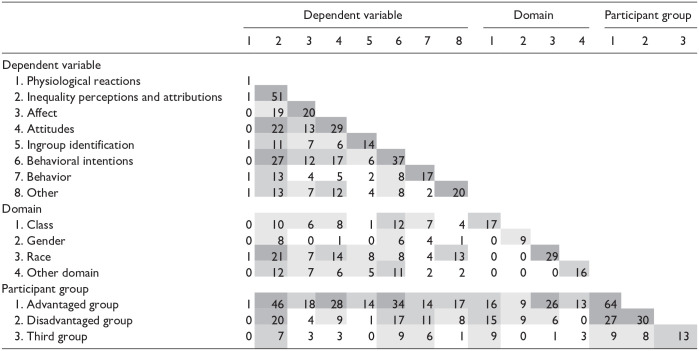

*Note.* The study space analysis shows overlap of investigated dependent variables, domains, and participant groups in included studies. For example, the second column shows that, out of 51 studies investigating inequality perception, 19 also looked at affect, 22 at attitudes etc., and that 10 were conducted in the domain of class inequality, 8 in gender inequality, 21 in racial inequality, and 12 in another domain. For another example, the ninth column shows that out of 17 studies investigating class inequality, 16 included participants from an advantaged group, 15 (also) included participants from a disadvantaged group, and 9 included participants from a third group.

Shading from light to dark = 0–5, 6–10, 11–20, >20 studies investigated this combination.

##### Investigated Contexts (RQ1c)

The effects of (dis)advantage framing have been investigated in various contexts, ranging from inequality between students of different universities to inequality between more or less affluent countries. However, there was a clear primary focus on racial inequality, followed by class, and gender inequality (the three domains together account for 77.5% of studies). Further, the studies were conducted in diverse countries. However, the vast majority was conducted in the United States or in Germany (84.6%), and the remaining in European countries, with only one exception (Nepal, with foreign tourists as participants, Greenaway et al., 2017). Thus, the investigated samples are almost exclusively restricted to WEIRD countries, with an overwhelming focus on the United States and Germany. Another problem is that the investigated domain varied with the country. For example, in the United States, the most-investigated domain was racial inequality; in Germany, it was class inequality.

The restriction of investigations of certain contexts to certain countries is critical because framing effects may vary with the sociocultural context. That is, sociocultural aspects may moderate (in)equality framing effects, for example, the size (Bruckmüller et al., 2017a) and perceived legitimacy of inequality (Harth et al., 2008), political attitudes (Chow & Galak, 2012), and the group membership of participants (Braun et al., 2023a). Sociocultural changes may even occur within a country, and thus account for fluctuating framing effects, given that perceptions of inequality can also change over time (Jun et al., 2022). Finally, most studies were conducted in English or German, and we identified none in a non-Romance and non-Germanic language. It is thus unclear whether (dis)advantage framing effects generalize across languages,

##### Types and Measurements of Dependent Variables (RQ1d)

The included studies investigated a rich variety of variables, ranging from perceptions and attributions of inequality, to behavioral intentions, attitudes, affect, behavior, ingroup identification, and physiological reactions. On the one hand, this variety is a strength of this relatively young field of research. On the other hand, this also means that effects have rarely been replicated in independent studies or across different inequality domains. For example, ingroup identification was relatively often examined for racial inequality but rarely for other domains. Further, most studies used scales, some open responses, but only a fraction measured (other) real behavior.

##### Summary of Methodological Scope (RQ1)

In sum, previous studies have examined many ways in which framing can affect recipients’ understanding of and reactions to inequality. Importantly, unsystematic variation in the methodology of previous studies may have contributed to the puzzle of sometimes contradictory findings, which we report in the following paragraphs.

#### Main Findings of Reviewed Studies (RQ2)

Below, we will summarize previously documented main effects of (dis)advantage framing (RQ2a) and identify (hidden) moderators of these effects (RQ2b). All reported main effects refer to a difference in the dependent variable between the disadvantage and the advantage framing condition (because the majority of studies did not have a control condition; see Supplemental Table B5).

Note that the included studies were not sufficiently comparable to aggregate effect sizes across dependent variables (too few studies per variable) or types of dependent variables (too many different variables per category). We report all unique effects in the supplement.^3^ The following paragraphs contain graphical and narrative summaries of (dis)advantage framing effects and are structured as follows. First, we will illustrate the inconsistency of earlier findings using the example of (dis)advantage framing effects on perceptions and attributions of inequality. Then, for all types of dependent variables, we will summarize only those effects of (dis)advantage framing, which were observed at least somewhat consistently across studies. Finally, we will attempt to identify hidden moderators, before giving a conclusive answer to RQ2.

##### An Example of Inconsistent Effects

**Figure 2.**
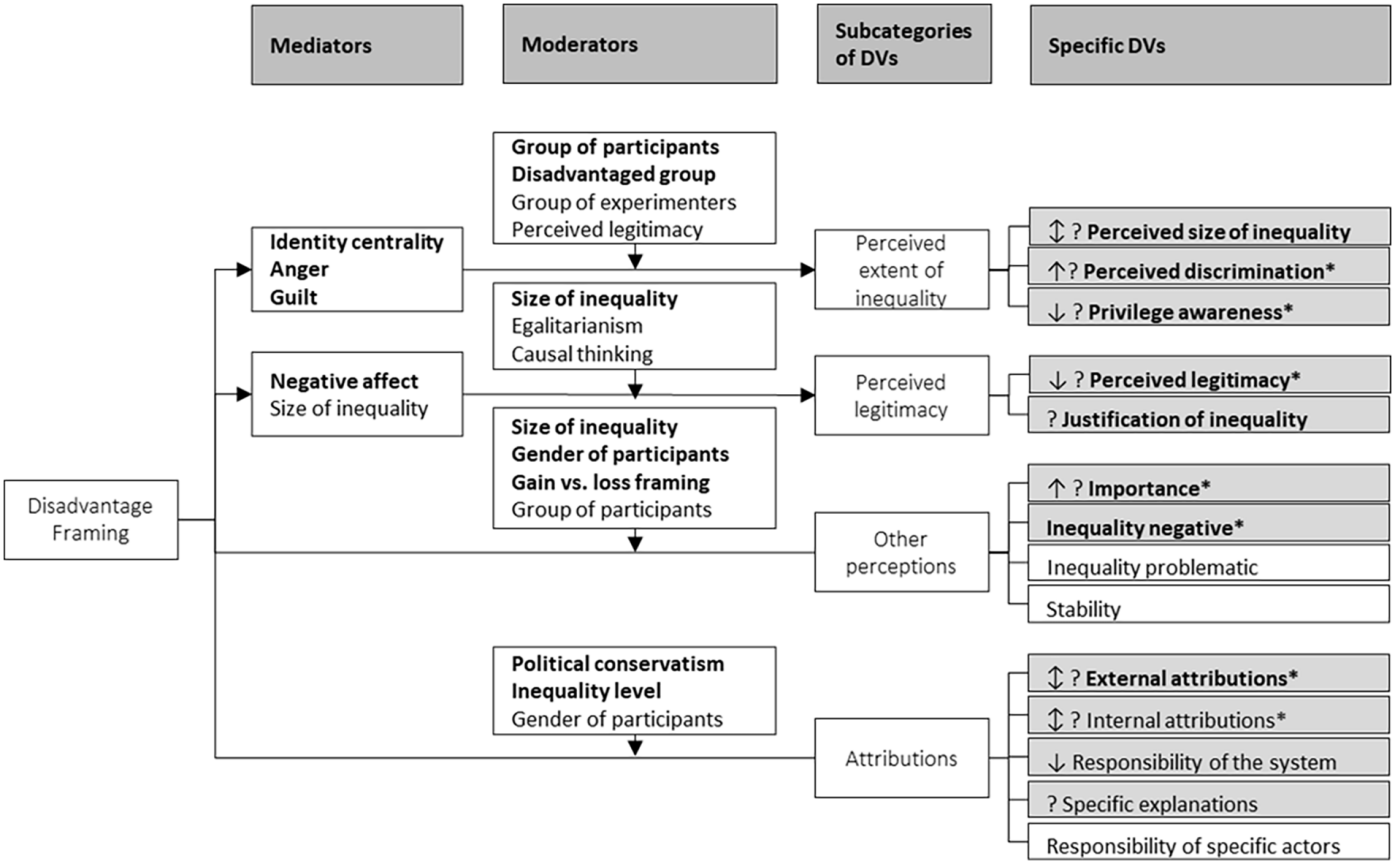
Effects of Disadvantage (Compared to Advantage) Framing on Perceptions and Attributions of Inequality. *Notes.* Hypothesized main effects, moderators, and mediators in **bold**. Gray versus white shading = *at least one* study (gray) versus *no* (white) study found a framing main effect on the dependent variable (DV). ↑ versus ↓ versus ↕ = main effect of disadvantage framing such that it led to *more* versus *less* versus to *more or less in different studies* of the DV, compared to advantage framing. ? = at least one study found and at least one failed to find a framing main effect on the DV. * = significant interaction of framing and moderator on DV. Interaction effects, moderators, and mediators that were not significant in at least one study are not depicted in this figure.

We will use perceptions and attributions of inequality as an example to illustrate the inconsistency of earlier findings because this was the most-investigated type of variable (see study space analysis). Figure 2 shows the broad range of effects, moderators, and mediators examined for perceptions and attributions.

###### Scattered and Inconsistent Findings

First, it is apparent that, for identical dependent variables, some studies found framing effects in contradictory directions, while others found them to depend on moderators, only found them for one inequality domain or one group of participants, or not at all. That is, findings were inconsistent across studies, and sometimes even contradictory (see Figure B1 for similar overviews for all other types of variables).

Consider the following examples. A *contradictory* finding was that disadvantage framing made gender inequality seem larger (Schnepf et al., 2023), but made class inequality seem smaller (Kraus et al., 2022; of course, this could also be an example for domain-specific findings). An example for the *dependency on moderators* was that disadvantage framing made inequality sometimes seem less legitimate (Greenaway et al., 2017), but others found that this was only the case when inequality was large (Bruckmüller et al., 2017a). An example for *domain-specific* effects is that disadvantage framing made gender inequality appear more important (Braun et al., 2023a), but not racial inequality (Ash & Schmierbach, 2013). Finally, an example for *inconsistent* findings was that the effect of (dis)advantage framing on the perceived size of class inequality did not replicate in later studies (Braun et al., 2023b).

###### Conceptual Gaps and Inconsistencies

Figure 2 also shows important conceptual gaps. For example, moderators were much more often investigated than mediators, and mediators were never experimentally manipulated. Finally, there were also conceptual inconsistencies, for example, regarding the role of central variables. Several variables were considered as both dependent variables and as moderators of framing effects (e.g., legitimacy is a dependent variable in Bruckmüller et al., 2017a, but a manipulated moderator in Salgado and Aguilar, 2022).

###### The Pattern for Other Dependent Variables

The pattern of inconsistent and sometimes contradictory effects was similar for all other types of variables (i.e., physiological reaction, affect, attitudes, ingroup identification, behavior[al intentions], and other variables; see Figure B1). To illustrate this scattered nature of findings, we briefly present examples across all types of variables. A *contradictory* finding was that disadvantage framing sometimes increased racial identification (Powell et al., 2005) and sometimes decreased it (Schröder, 2021). An example for the *dependency on moderators* is that advantage framing sometimes increased collective guilt among the advantaged (Powell et al., 2005), but others showed that this was only the case when inequality was illegitimate (Harth et al., 2008). An *inconsistent* effect was that disadvantage framing increased modern racism in the studies by Powell et al. (2005), but this did not replicate in later studies (e.g., Ash & Schmierbach, 2013; Estrada, 2012).

An additional observation we made was that there were many effects which were investigated only once, that is, which were never replicated. One example is effects on physiological reactions (Dover, 2022). Another is different emotions which were measured only once, such as shame in Estrada (2012), sympathy in Harth (2007), or gratitude in Puryear et al. (2019). Other effects were examined several times by the same authors, but never by others (e.g., feelings of sympathy and pride, Harth, 2007; causal explanations for inequality in open responses, Braun et al., 2023a, Bruckmüller & Braun, 2020).

In sum, our first response to RQ2 is that previous research has documented a rich variety of (dis)advantage framing effects, which were often only investigated once, or which were inconsistent, or even contradictory. Despite this, we below give a narrative account of those effects that appear consistent to at least some extent.

##### Consistent (Dis)Advantage Framing Effects (RQ2a)

The following summary of (dis)advantage framing effects is ordered by type of dependent variable. We equally include findings from peer-reviewed, non-peer reviewed, and unpublished manuscripts.^4^ For each type of dependent variables, to identify (at least somewhat) consistent effects, we include those which were pre-registered because these provide a more reliable estimate of true effects (Schäfer & Schwarz, 2019). We report findings for all pre-registered effects, including those that were not significant. Additionally, we include all effects which were hypothesized and found in at least two studies (including, but not limited to, studies within the same paper). We wanted to include as many effects as possible and considered this to be the absolute minimum threshold for consistent effects. This low threshold allowed us to give justice to the range of potential (dis)advantage framing effects, while not giving undue weight to never-replicated, preliminary evidence. To also make inconsistent findings transparent, for each effect that we include as (at least somewhat) consistent, we always give examples for studies which found or failed to find the respective effects, if they exist.

##### Physiological Reactions

There were no effects that fit the above criteria.

##### Perceptions and Attributions of Inequality

(Dis)advantage framing can affect the perceived extent, legitimacy, attributions, and other perceptions of inequality. Disadvantage framing leads people to perceive inequality as larger (Brown & Craig, 2020; Schnepf et al., 2023). However, it can also lead the advantaged to defensively point out personally faced discrimination and to be less aware of their privilege (Brown & Craig, 2020; Schröder, 2021). Disadvantage framing can make inequality seem less legitimate (Dietze & Craig, 2021), but this is not always the case (pre-registered: Braun et al., 2023b; not pre-registered: Bruckmüller et al., 2023; Dover, 2017; Schnepf et al., 2023, 2021; Yanar, 2018). One explanation for this could be that perceived size of inequality moderates the effect on legitimacy. Dietze and Craig (2021) investigated reactions to rather large differences, and several studies found that disadvantage framing only affected legitimacy when inequality was (perceived as) large (Bruckmüller et al., 2017b, 2017a; not replicated by Schnepf et al., 2021). One pre-registered study failed to replicate a finding (from the same manuscript) that framing would interact with participants’ group membership in predicting internal attributions of gender inequality (Bruckmüller & Braun, 2020). One pre-registered study found that perceived size of inequality was a stronger predictor of negative evaluations of inequality after disadvantage than after advantage framing (Bruckmüller et al., 2017b).

##### Affect

Advantage framing can sometimes induce collective guilt among the advantaged (Powell et al., 2005; not replicated by Estrada, 2012; Greenaway et al., 2017; pre-registered: Schröder, 2021). One explanation for inconsistent findings is that advantage only induces guilt or negative affect when inequality is perceived as illegitimate (Harth, 2007) or as large (Schnepf et al., 2021). The advantaged may also feel proud about legitimate ingroup advantage (mixed evidence: Harth, 2007). Further, disadvantage framing can lead to more feelings of moral outrage (Greenaway et al., 2017; inconsistent: Cervone et al., n.d), and to more sympathy toward the disadvantaged group (Hart, 2007).

##### Attitudes

Disadvantage framing may lead to worse intergroup attitudes (stereotyping: Bruckmüller et al., 2012; racism: Powell et al., 2005; Schröder, 2021), although the evidence on this is mixed (racism: Ash & Schmierbach, 2013; Estrada, 2021; intergroup attitudes: Greenaway et al., 2017; stereotyping, pre-registered: Malapally & Bruckmüller, 2022). There is also preliminary, but mixed evidence that disadvantage framing may lead to a higher perceived need for interventions (pre-registered: Braun et al., 2023b; Bruckmüller et al., 2023).

##### Ingroup Identification

Advantage framing can sometimes lead the advantaged to identify less with their group, at least in some facets (ingroup esteem, Lowery et al., 2012, identity centrality, pre-registered: self-investment; Schröder, 2021), and possibly only among people with a high preference for meritocracy (Chow et al., 2008). However, the evidence for this is mixed (global identification: Chow et al., 2008; Dover, 2017; identity centrality, self-stereotyping: Dover, 2017; numerous other facets: Schröder, 2021).

##### Behavioral Intentions

Disadvantage framing may increase collective action support (pre-registered: Dietze & Craig, 2021), but the evidence for other, specific interventions is mixed (pre-registered: Braun et al., 2023b; Bruckmüller & Braun, 2020; Malapally & Bruckmüller, 2022; not pre-registered: Ash & Schmierbach, 2013; Bruckmüller et al., 2023; Schnepf et al., 2023). There is some evidence that participants support interventions more when inequality is perceived as large (Schnepf et al., 2023) and when they are framed in the same way as the inequality they aim to reduce (e.g., targeting White people to reduce White advantages: Lowery et al., 2012; not replicated by Valeri & Burgeson, 2007). (Dis)advantage framing effects on behavioral intentions can also be moderated by inequity level (inequality between individuals versus group-based; Rosette & Koval, 2018) and perceived legitimacy (Harth, 2007; not replicated by Salgado & Aguilar, 2022).

##### Communication Behavior and Other Behavior

Disadvantage framing leads participants to focus explanations of and interventions against inequality more on the disadvantaged group (pre-registered: Braun et al., 2023b; Bruckmüller & Braun, 2020; not replicated for explanations by Yanar, 2018). Looking at the content of explanations, however, framing did not affect internal attributions in explanations of gender inequality (pre-registered: Bruckmüller & Braun, 2020). Disadvantage framing may increase user engagement on social media (Dietze & Craig, 2021).

##### Other Variables

Disadvantage framing can make the disadvantaged disengage from academic outcomes, that is, weaken the effect of performance on self-esteem (Lowery & Wout, 2010).

Taken together, in response to RQ2a, (dis)advantage framing can affect how people perceive and react to inequality in many ways, but the precise effects depend on moderators. Importantly, some of these moderators can also be influenced by framing (e.g., size, legitimacy of inequality), making it hard to draw overall conclusions. Given the inconsistencies of earlier research, we suspected that there are other, potentially hidden moderators.

##### Hidden Moderators (RQ2b)

One hidden moderator may be *inequality domain*. For example, disadvantage framing made class and global inequality seem less legitimate (e.g., Dietze & Craig, 2021), but not racial and gender inequality (e.g., Dover, 2017; Schnepf et al., 2023). Disadvantage framing led to more stereotyping of women and men (Bruckmüller et al., 2012), but not Black and White people (Walker, 2013). This may be due to different habits around framing different domains. Racial and, a bit less so, gender inequality are mostly framed as disadvantage, but class inequality is most often framed with no particular focus or as advantage (at least in the United States; Jun et al., 2022). Arguably, framing varies between inequalities because there are different norms about discussing them (Dover, 2022), and disadvantage framing may be more common for more illegitimate inequalities (e.g., racial inequality, Jun et al., 2022), and less common for inequalities conceptualized as differences in positive rather than in negative outcomes (e.g., wealth inequality; Malapally & Bruckmüller, 2024). Importantly, the dimensions in which inequalities differ from each other (e.g., legitimacy, size) are moderators of (dis)advantage framing, so domain itself is likely also a moderator. Considering domain as a moderator is useful because it combines several important features of inequality (e.g., legitimacy, size), which in practice can hardly be viewed as independent from domain, thus enabling meaningful practical implications.

Another possible moderator is a *recipient’s group membership*. (Dis)advantaged groups may react differently to inequality (frames) because they are differently affected by them (Branscombe, 1998). Some framing effects mirror for disadvantaged versus advantaged groups. For example, while disadvantage framing made disadvantaged participants disengage from academic outcomes, advantage framing led advantaged participants to disengage (Lowery & Wout, 2010). Other findings do not mirror. For instance, disadvantage framing made lower and upper class participants support collective action more (Dietze & Craig, 2021). Importantly, even if a frame evokes similar responses for different groups, it may do so through different pathways. While disadvantage framing evokes moral outrage regardless of participants’ group membership (Cervone et al., n.d.), it presumably only evokes more sympathy among the advantaged (Harth, 2007), and both emotions may motivate collective action on behalf of the disadvantaged group.

In sum, in response to RQ2b, we identified inequality domain and recipients’ group membership as hidden moderators of (dis)advantage framing effects.

##### Summary of Main Findings (RQ2)

In conclusion, (dis)advantage framing effects were largely inconsistent across studies, with some finding no effects, and others finding effects in contradicting directions for identical or highly similar dependent variables. Nevertheless, we identified some relatively consistent effects. Disadvantage framing can make inequality seem larger and less legitimate, while advantage framing can increase privilege awareness. Framing can also elicit specific emotions, such as moral outrage and sympathy in reaction to disadvantage framing, while advantage framing can elicit guilt. Disadvantage framing can worsen intergroup attitudes. Advantage framing can lead to lower ingroup identification among the advantaged. (Dis)advantage framing can also guide people to focus explanations of and solutions for inequality more on the (dis)advantaged group. Finally, disadvantage framing can increase support for collective action. However, almost all these effects depended on moderators (such as the size and legitimacy of inequality, or the attitudes of recipients), and they could not always be replicated. Additionally, we identified the group membership of recipients and the inequality domain as hidden moderators of (dis)advantage framing effects. Below, we analyze the theoretical foundations of previous studies, before drawing overall conclusions from the systematic review.

#### Theories Used to Explain (Dis)Advantage Framing Effects (RQ3)

We analyzed which theories were used in previous research to explain (dis)advantage framing effects. First, raters coded which theory was mentioned, either by name (e.g., “norm theory”) or by describing key concepts and citing the authors (i.e., that mental category norms guide recipients to focus explanations of differences within a category on less normative category members because they become more salient; Kahneman & Miller, 1986; Miller et al., 1991). Then, raters coded whether this theory was used explicitly to derive hypotheses or not.

We also analyzed to what extent the theories were used to explain (dis)advantage framing effects. A broad range of theories appeared in the manuscripts, including social identity theory, focalism, norm theory, prospect theory, emphasis framing, as well as 12 other theories (e.g., relative deprivation theory, Harth, 2007; inequality framing model, Littleford & Jones, 2017; social dominance theory, Lowery et al., 2012; equity theory, Valeri & Burgeson, 2007). We will describe the theories and what predictions they make in more detail below.

**Figure 3.**
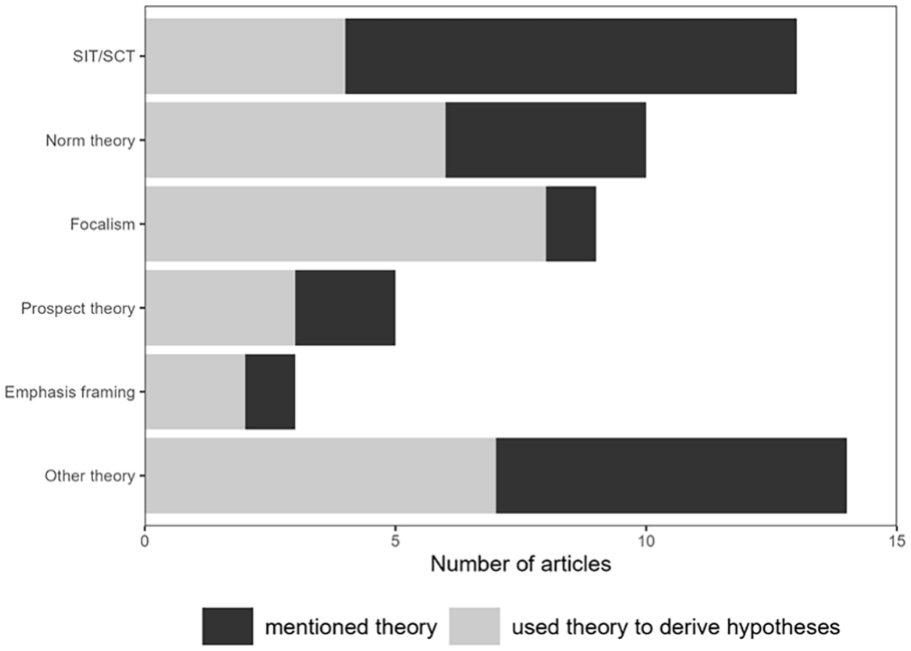
Number of Articles Which Mentioned and the Subset Which Used Particular Theories to Derive Hypotheses. *Note.* SIT = social identity theory. SCT = self-categorization theory. Several manuscripts mentioned more than one theory. In seven manuscripts, no specific theory was mentioned to explain framing effects.

Many of these theories, although mentioned in the introductions, were not used explicitly to derive hypotheses on the effects of (dis)advantage framing (see Figure 3). Further, framing effects were sometimes not explained by any specific (previously established or newly developed) theory, and hypotheses were solely based on previous findings (*n* = 7). For one unpublished manuscript, only method and results were available.

In sum, previous studies used many different theories to explain (dis)advantage framing effects, but did not always use them to derive hypotheses, sometimes only basing them on previous empirical findings. Thus, previous research does not seem to be guided by a common theoretical approach and some of it even appears to not be guided explicitly by theory. This, again, points at the need to integrate future research on conceptual level.

### Main Conclusions from the Systematic Review

The systematic literature review revealed that previous studies vary unsystematically in which domains, groups of participants, and outcomes of (dis)advantage framing they investigated and left us with a puzzle of inconsistent, and sometimes contradictory, findings. However, it allowed us to identify some likely hidden moderators. The review also documented the broad range of theories used previously to explain (dis)advantage framing effects as well as, for part of the literature, a certain gap between cited theories and specific predictions. Thus, the theoretical basis of (dis)advantage framing effects seems to be just as scattered as its empirical one. It even seems plausible that the lack of integrating theory is one *reason* for the jumble of empirical findings. In the following, we therefore aim to integrate previous theoretical and empirical work on a conceptual level.

## Toward Conceptual Integration: A Two-Step Model of (Dis)Advantage Framing Effects

Previous research has documented the diverse ways in which (dis)advantage framing can affect responses to inequality, but these empirical findings are often inconsistent, not replicated, or even contradictory. Further, there is no comprehensive theorizing explaining why and how these effects occur. The latter aspect is not surprising given that research on (dis)advantage framing effects is a relatively young field. Emerging research is often characterized by loosely related theoretical perspectives (Elsbach & Van Knippenberg, 2020). Yet, for a field at this stage, it is an imperative next step to consolidate existing knowledge, and to advance further research through conceptual integration. Such integration also enables the development of comprehensive, future research agendas (Cronin & George, 2023). Our goal here is to take this step.

Thus, in the following, we identify a core common denominator in previous theoretical approaches that allows us to integrate these various approaches in one overarching conceptual model. Through this integration, we aim to answer some of the most pressing open questions in this research field, including why (dis)advantage framing can affect which types of responses, which moderators should be considered, and which constructs have been neglected so far. This integration also helps to identify research gaps and to derive new, testable predictions, thus crafting a future research agenda in a more systematic way. In short, we will show how far research on the effects of (dis)advantage framing has come and where we propose it should go next.

### Previous Theories on (Dis)Advantage Framing Effects

#### Inequality Framing Model/Inequity Frames Theory

We are aware of one previous attempt to integrate the literature on (dis)advantage framing effects. Phillips and colleagues (2022) analyzed the extent to which advantage and disadvantage frames are used in scholarly work on gender and racial inequity. In their justification for why these frames matter, they provided a narrative review of previous research on the effects of (dis)advantage framing which, however, remained relatively brief and somewhat selective, given that it was not the main focus of the respective publication. The theoretical model they suggest to explain these effects is *Inequity Frames Theory* (IFT, Phillips et al., 2022), which together with the closely related *Inequality Framing Model* (IFM, proposed earlier by partially overlapping authors, Chow et al., 2010) is the only theoretical model (or pair of models) specifically developed for (dis)advantage framing effects that we could identify in our review. It states that (1) inequality can be framed either as disadvantage or as advantage and (2) that “each frame leads to different sensemaking responses” (Phillips et al., 2022, p. 550). While this provides an overarching conceptualization of (dis)advantage framing effects and why they occur, the theory/model does not provide an explicit definition of (dis)advantage framing and it remains relatively unspecific with regard to the psychological processes that result from it, although one specific example of different sensemaking processes is provided, namely that depending on the chosen frame, one group becomes the normative or equity standard which the other group is compared to. This means that people do not just consider the relative differences between groups, but compare the status of a respective group to an assumed standard of what people should have (see also Lowery et al., 2007). Thus, IFT and IFM, while taking important first steps toward providing a conceptual model for (dis)advantage framing effects, cannot fully explain why such framing effects occur or make specific predictions for moderating processes or boundary conditions. Consequently, in the studies we systematically reviewed above, both models were not explicitly employed to make predictions about reactions to (dis)advantage framing, and only once the IFM was mentioned to explain (dis)advantage framing effects (Littleford & Jones, 2017). Instead, diverse psychological processes and theories have been used to predict and explain (dis)advantage framing effects, of which we give a few examples below.

##### Focalism

The principle of *focalism* entails that the target of a comparison becomes more salient and is drawn on more in subsequent processing than the referent (Chambers & Windschitl, 2004; Tversky, 1977). For example, disadvantage framing of class inequality (“the poor have less than the rich”) draws more attention to the poor than to the rich. The target (the poor) and its characteristics should be more accessible and drive perceivers’ reactions to the issue more than characteristics of the referent (the rich). Some studies based their hypotheses on focalism explicitly (e.g., Braun et al., 2023a; Bruckmüller & Braun, 2020; Schnepf et al., 2021). For example, Schnepf et al. (2021) argued that disadvantage framing makes inequality appear less legitimate than advantage framing because the former makes mental representations of poverty salient and accentuates negative features of inequality, while the latter accentuates wealth and positive features. Other research draws on the principle of focalism implicitly, by referring to a similar mechanism without referencing focalism (e.g., “Compared to [. . .] out-group disadvantage [. . .], ingroup privilege highlights an aspect of the ingroup that typically goes unnoticed,” Powell et al., 2005, p. 510).

##### Norm Theory

IFT/IFM’s suggestion that (dis)advantage frames cause one group to become the normative standard to which the other group is compared, as well as focalism’s emphasis on the target’s salience in comparisons, is conceptually similar to *norm theory* (Kahneman & Miller, 1986; Miller et al., 1991). This theory argues that upon encountering a category label or stimuli belonging to a certain category, people create an implicit mental norm for that category, which serves as a frame of reference for further judgment. This matters for comparisons between social groups (and therefore inequality framing) as these implicit mental norms cause perceivers to focus explanations of differences within a category on less normative category members because their deviation from the norm makes them particularly salient (see Hegarty & Pratto, 2001). At the same time, the way intergroup differences are framed (who is compared to whom) implicitly communicates normativity and deviation (Hegarty & Bruckmüller, 2013). (Dis)advantage framing establishes normativity and deviation by making one group the referent (rendering it the normative standard) and one the target that is compared to this standard (making it more salient). This in turn affects responses to inequality (e.g., Lee, 2018). For example, norm theory has been used to explain why (dis)advantage framing can affect how legitimate people find economic inequality. Advantage framing (“the rich have more than the poor”) renders the poor the normative standard, thus implying that wealth is a positive deviation from a more modest financial standard. In contrast, disadvantage framing (“the poor have less than the rich”) makes poverty seem like a negative deviation from a more affluent standard. Thus, disadvantage framing should make (large) inequality seem less legitimate compared to advantage framing (Bruckmüller et al., 2017a).

##### Prospect Theory

*Prospect theory* posits that individuals are more motivated to act when faced with the threat of loss than when faced with the opportunity for gain. Individuals, therefore, weigh negative aspects more heavily than positive ones (Kahneman & Tversky, 1979). By choosing one group as the referent, inequality can either be framed in positive (e.g., “men earn more than women”) or negative terms (“women earn less than men”) which can evoke specific responses (e.g., Ash & Schmierbach, 2013; Bruckmüller & Braun, 2020). For example, prospect theory may explain why (dis)advantage framing can affect support for collective action. Since disadvantage framing makes people focus on the negative side of inequality, and negative information is weighed more, this should create more motivation to reduce inequality and, hence, more willingness to act (Bruckmüller & Braun, 2020).

##### Social Identity/Self-Categorization Theory

*Social identity theory* (SIT, Tajfel & Turner, 1979) and *self-categorization theory* (SCT, Turner et al., 1987) posit that social groups and their standing are an important part of self-definition, and that individuals can assess their group’s status through social comparison. Since (dis)advantage framing implies upward versus downward comparisons, these frames draw attention to the inferior or superior position of the in- and outgroup and should thus differentially affect participants’ responses (e.g., Powell et al., 2005; Schröder, 2021). For example, social identity theory has been used to explain the effect of (dis)advantage framing on ingroup identification. Since advantage framing can highlight the unearned benefits of one’s ingroup, this can undercut pride in this identity and, thus, reduce ingroup identification (Powell et al., 2005).

#### What Distinguishes and What Connects All These Theories?

Taken together, focalism, norm theory, prospect theory, and SIT/SCT all provide predictions for specific kinds of different psychological processes that occur in response to disadvantage versus advantage framing (as summarized by IFT/IFM). Yet, they differ in why and how they propose this happens. In focalism, the key process is higher cognitive accessibility of the target’s characteristics (Chambers & Windschitl, 2004; Tversky, 1977). In norm theory, it is the higher implicit normativity of the referent group (Kahneman & Miller, 1986; Miller et al., 1991). In prospect theory, the key driver of framing effects is different sensitivity to gains and losses, or positive and negative information more generally (Kahneman & Tversky, 1979). In social identity theory, the key ingredient is increased attention to the superior or inferior position of the in- or outgroup (Tajfel & Turner, 1979). As a result of the inclusion of different key mechanisms, each of the above theories has been used to explain different consequences of (dis)advantage framing. For example, norm theory and focalism have been used to explain why these frames can affect legitimacy appraisals, prospect theory to explain effects on collective action support, and social identity theory to explain effects on ingroup identification.

Yet, despite their differences, there is an important commonality between the different theoretical approaches, namely, that they all suggest that framing puts one aspect (i.e., advantage or disadvantage) of an issue (i.e., inequality), or one involved group (i.e., the ingroup or the outgroup) in the foreground, while masking others. The different theories then zoom in on different consequences of this foregrounding. Nevertheless, they all entail the assumption that (dis)advantage framing makes (dis)advantages and/or the (dis)advantaged group, or particular characteristics of this group, more salient than the respective other outcomes, groups, and/or group characteristics. This is, incidentally, the basic tenet of focalism (Chambers & Windschitl, 2004; Tversky, 1977). We, therefore, suggest that focalism offers a unifying lens that allows an integration of different theoretical approaches.

Importantly, however, focalism in and of itself does not capture many of the unique predictions made by other approaches, for example, of SIT/SCT’s predictions for effects on intergroup emotions and ingroup identification. Thus, further considerations are needed on how these approaches can be integrated under the premise of framing inducing focalism. In the following, we will use the insight of focalism as a commonality in previous approaches to build an overarching, conceptual framework for understanding (dis)advantage framing effects that allows an integration of the unique predictions and insights produced by various previous theoretical approaches to inequality framing. Beforehand, we will give a short outlook of the aims and scope of the proposed model.

### Aims and Scope of the Proposed Two-Step Model

With our model, we aim to achieve several goals. First, we provide an explicit definition of (dis)advantage framing, which is rooted in framing theory. Above, we have identified this as a major theoretical gap in previous theorizing. Second, we aim to provide an explicit rationale of *why* and *how* (dis)advantage framing affects *which* variables.

**Figure 4.**
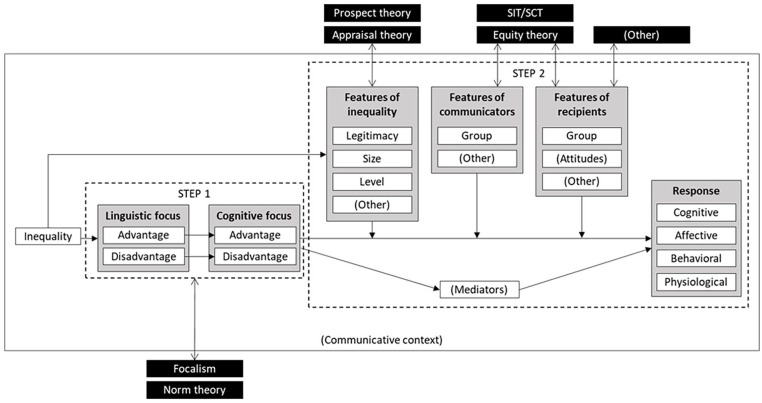
The Two-Step Model of (Dis)Advantage Framing Effects. *Note.* In the first step, the linguistic (comparative) focus is translated to a cognitive focus on either advantage or disadvantage, and predictions for how this happens can be deducted from focalism and norm theory. In the second step, the cognitive focus affects recipients’ responses to inequality and these reactions are moderated by features of the inequality, the communicators, and the recipients. This links the model to further exemplary theories, which in turn explain the *specific* responses. Boxes in black show exemplary links to established theories. Variables in (brackets) show research gaps to be addressed by future research.

To do this, we divide reactions to (dis)advantage framing into two steps (see Figure 4). The first step is cognitive. We posit that the specific framing guides more attention to one aspect of inequality (disadvantage or advantage), and less to the other. This step is the overarching common denominator that allows an integration of various different approaches in previous work. The second step comprises cognitive, affective, behavioral, and physiological responses. We posit that these responses depend on the features of the inequality, the communicators, the recipients, and the overarching context. This second step is what allows us to integrate the unique insights that specific theoretical approaches offer for specific effects of (dis)advantage framing. In this second step, we also systematize dependent variables and moderators in superordinate categories, including those that have previously been researched as well as potential other variables and moderators. This serves to reconcile previous inconsistent findings, and to craft directions for future research (including both the identification of research gaps and the deduction of novel predictions). Taken together, we propose a conceptual model that integrates and advances previous theorizing, systematizes existing empirical findings, and provides innovative directions for future research.

At this point, some boundaries of the proposed model need to be made explicit. The scope of the two-step model covers *recipients’ reactions* to (dis)advantage frames of *social* inequality (i.e., between social groups). Thus, the choice of these frames by communicators and inequalities between individuals (without reference to their social group) are *not* within its scope. Further, the model is restricted to languages which allow (dis)advantage framing, that is, where comparisons are constructed with a target and a referent (see below). Finally, the model will not result in *general* predictions regarding the effects of (dis)advantage framing on recipients’ perceptions of and reactions to inequality, as we believe these to be impossible due to the complexity and context-dependency of (dis)advantage framing effects (see below). This also makes clear that the question of which framing is better suited to challenge inequality, although tempting to ask from a practical standpoint, cannot be answered on a general level. Instead, we will bring together previous theorizing and empirical findings in one integrative conceptual model, which can then be modified and applied to specific research questions (or questions concerning practical implementations), to derive specific predictions, contingent on specific moderators (we will make specific examples for how this can be done).

In the following section, we will first give an explicit definition of (dis)advantage framing. Then, we will detail the two steps and substantiate them with empirical evidence. Finally, we will outline potential applications in research practice and some new predictions the model inspires, using concrete examples.

### Defining (Dis)Advantage Framing

We posit that an explicit definition of (dis)advantage framing should be rooted in framing theory and be related to established types of framing, such as equivalency or emphasis framing (Cacciatore et al., 2016; Druckman, 2004). Following Scheufele and Iyengar (2014), we suggest a conceptual narrowing of (dis)advantage framing to variations of *how* inequality is presented (i.e., framed) and not *what* is communicated. Thus, we here define (dis)advantage framing as logically equivalent variations (i.e., equivalency framing) of describing inequality between two social groups as either advantage of one group or disadvantage of another group via the focus of comparison. In line with the proposed definition, some earlier studies varied only the comparison focus (with otherwise parallel wording). Other studies also varied terms such as “privilege” or “discrimination.” While the effects of such terms are of course a legitimate research question in their own right, a narrow definition of (dis)advantage framing aims to prevent confounding effects of equivalency frames with other informational and persuasive features of messages that are present in emphasis frames (Scheufele & Iyengar, 2014). Relegating research on the effects of specific terms to studies beyond (dis)advantage framing in a strict sense or to conceptually separate equivalency and emphasis frames (e.g., by independently manipulating them) would certainly help to enhance conceptual clarity. Following the definition of (dis)advantage framing, the next task is to lay out the two steps in which it affects recipients’ responses.

### The Two Steps of (Dis)Advantage Framing Effects

Above, we have identified the focus on either disadvantage or advantage as a commonality of previous explanations of (dis)advantage framing effects. In the first step of the proposed model, we differentiate this focus further, into the linguistic focus on either aspect of inequality (which is a characteristic of the framed information) and the cognitive focus (which is induced in recipients).

#### From Linguistic to Cognitive Focus (Step 1)

To develop the first step of our model, we will first define the linguistic and the cognitive focus and explain how they have been operationalized. Then, we will describe how the linguistic focus is translated into the cognitive focus, which is the process that constitutes Step 1.

##### Linguistic Focus

(Dis)Advantage framing can be implemented via or determined through (grammatical) analysis of the *linguistic focus* of a message. This can be done manually or automatically, the latter enabling analysis of large text corpora (e.g., Malapally et al., 2024). An example for a disadvantage frame is saying that the poor have less than the rich. This statement linguistically positions the disadvantaged group as the *target* (or focus) of a comparison (here: the poor) and the advantaged group as the *referent* that this target is compared to (the rich). In short, it compares the disadvantaged group to the advantaged group. An advantage frame (e.g., the rich have more than the poor) positions the advantaged group as the target (the rich), and the disadvantaged group as the referent (the poor). In short, it compares the advantaged to the disadvantaged group.

##### Cognitive Focus

The cognitive focus is the selective attention to a salient aspect of social inequality, that is, disadvantage versus advantage, or the disadvantaged versus advantaged group. In previous research, cognitive focus has been measured (implicitly) by inference from individuals’ framing choices. For example, researchers have presented disadvantage or advantage frames to participants and then asked them to explain or describe inequality in their own words. Then, they analyzed which frames (Braun et al., 2023a) and which first- or third-person pronouns participants used in their responses (Powell et al., 2005). We discuss in the future research section how the cognitive focus could be measured more directly.

##### The Linguistic Focus Induces a Cognitive Focus

The first core proposition of our model is that when recipients are exposed to social inequality framed as either disadvantage or advantage, the linguistic focus shapes what is salient to recipients, that is, the linguistic focus induces a parallel cognitive focus on either disadvantage or advantage (Step 1, the left part of Figure 4). This proposition draws primarily on focalism and norm theory, which both explain why the linguistic focus can induce a cognitive focus on either disadvantage or advantage (both approaches are, therefore, linked to Step 1 in Figure 4). Focalism explains (dis)advantage framing effects by a higher salience of the target of a comparison and its characteristics (relative to the referent and its characteristics). Norm theory instead focuses more on the contrast between the target and the referent and allows predictions of which aspects of the target will become particularly salient, namely, those that distinguish it from the referent. Thus, combining both approaches allows rather clear predictions for how linguistic focus shapes what does and does not become salient for recipients (i.e., the cognitive focus). However, while this salience explains in principle *why* (dis)advantage frames lead to different responses to social inequality, it cannot predict what these responses look like (i.e., *how* [dis]advantage framing affects different outcome variables). For this, we need to consider further processes and the moderators that affect them. This is what Step 2 of the model does.

#### From Cognitive Focus to Specific Responses (Step 2)

In Step 2, the focus on disadvantage or advantage is translated into specific responses. The core proposition here is that the cognitive focus on either disadvantage or advantage guides subsequent processing and reactions (the right side of Figure 4), and that these are contingent on various moderators. These moderators can vary depending on the specific context of inequality and the outcome variable of interest. For example, it seems obvious that the cognitive focus on either advantage or disadvantage can evoke very different emotional reactions in a recipient depending on whether they belong to a disadvantaged or an advantaged group, how legitimate they find the inequality, and who confronts them with it. For example, a privileged person may feel proud about a legitimate advantage, while a disadvantaged person may feel anger about illegitimate disadvantage. This shows that Step 2 is necessary to derive predictions for *how* (dis)advantage framing affects *which* dependent variables and *which* moderators need to be considered in a particular context. In the following, we first categorize dependent variables and moderators on a conceptual level, before we link them to theories, using concrete examples.

##### Categorization of Dependent Variables

As illustrated in Figure 4, we propose to categorize dependent variables as cognitive (e.g., perceived size of inequality; Kraus et al., 2022), affective (e.g., guilt; Harth et al., 2008), behavioral (including behavioral intentions; for example, collective action support, Dietze & Craig, 2021), and physiological (e.g., cardiovascular threat, Dover, 2022), at least for now. This also covers more complex responses, such as ingroup identification (e.g., Lowery et al., 2012) or attitudes (e.g., racism; Powell et al., 2005), which combine several of these components, for example, attitudes that can be formed and expressed by cognitions and affect as well as behavior (Eagly & Chaiken, 2007).^5^ All dependent variables studied in previous research are covered by this classification, but additional categories could to be added to this list later.

##### Categorization of Moderators

We propose to classify moderators as features of the inequality (which are shaped by the immediate communicative and the overarching societal context) and inter-individual features (i.e., of communicators, recipients, and the relationship between them), see Figure 4. The most consistent moderators identified in our review were features of the inequality. In particular, perceived size of inequality moderated framing effects on perceptions of inequality (e.g., Bruckmüller et al., 2017a), legitimacy moderated effects on affect (e.g., Harth et al., 2008), and inequality level (inequality between individuals versus group-based) moderated effects on behavioral intentions (Rosette & Koval, 2018). We identified group membership of both communicators and recipients as the most notable inter-individual moderator. First, it matters whether the communicator is a member of the advantaged, disadvantaged, or a third group. For example, advantage framing induced guilt among advantaged participants, but only when delivered by an ingroup member (vs. an outgroup member; Greenaway et al., 2017) – arguably, because in the specific context of this study (a Nepali or foreign experimenter approaching foreign tourists in Nepal), this made group identity salient. Second, it also matters which group the recipient belongs to. Although few studies explicitly tested a recipient’s group membership as moderator (Lowery & Wout, 2010; Schnepf et al., 2023), disadvantaged and advantaged groups clearly differed in their responses across studies (see examples in the systematic review).

In sum, Step 2 provides a classification of the types of variables that can be influenced by (dis)advantage frames and of variables that may moderate these effects. Step 2 also links these moderators to established theories, thereby providing theoretical integration of previous research.

##### Links to Established Theories

Through the moderators at the level of inequality (e.g., size and legitimacy), the two-step model is linked, for example, to prospect theory and equity theory. Prospect theory can explain why size of inequality is a moderator. Small gains (or advantages) and losses (disadvantages) are perceived as relatively similar in value, while large losses are judged as more severe than large gains (Kahneman & Tversky, 1979). It thus follows that people react differently to the size of inequality when framed as advantage versus as disadvantage. A second example is equity theory, which predicts feelings of distress for unequal ratios of inputs to outcomes, that is, when inequality is illegitimate (Walster et al., 1978). This explains why people react differently to legitimate versus illegitimate (dis)advantage.

Further, through the moderators at the level of communicators and recipients (e.g., group membership), the two-step model is linked, for example, to SIT/SCT and appraisal theories of emotion. SCT suggests that highlighting the (dis)advantages of the ingroup should facilitate self-categorization at the group level more than highlighting the (dis)advantages of an outgroup (Turner et al., 1987). This theory thus predicts that disadvantaged and advantaged groups react differently to (dis)advantage framing. Group membership also links the two-step model to appraisal theories of emotions (see also Harth et al., 2008). They posit that individuals first appraise whether a situation affects the self or others, and then react (e.g., Moors et al., 2013). It follows, again, that members of (dis)advantaged groups react differently to different inequality frames.

#### The Communicative Context

Finally, the two-step model posits that (dis)advantage framing effects are moderated by features of the communicative context. For example, the socioeconomic and political context may influence perceptions of inequality, for example, how large or how legitimate participants find a presented inequality, and, as the systematic review revealed, these perceptions can moderate framing effects (Bruckmüller et al, 2017a; Harth et al, 2008). Different contexts may also evoke different cultural and linguistic norms, which could influence framing effects (Dover, 2022) – an idea suggested by some, but rarely empirically tested in previous research. One exception is that Bruckmüller et al. (2012) compared (dis)advantage framing effects between a normatively male context (leadership) and an undifferentiated context (leisure time). Only in the leadership context was gender inequality perceived as more legitimate when framed as disadvantage, compared to advantage (among other effects).^6^ This suggests that (dis)advantage framing effects may be context-dependent. In contexts where one group is the cultural default (here: men in leadership), this group usually also becomes the linguistic default (here: a male norm, i.e., disadvantage framing), leading to the problematic consequence of reinforcing inequality (here: higher legitimacy of gender inequality; see also Pratto et al., 2007). In sum, it seems plausible that the context moderates (dis)advantage framing effects (see also the construct of *cultural resonance* in emphasis framing research, for example, Tewksbury & Scheufele, 2019). However, since this possibility has never been systematically investigated, the communicative context is included in Figure 4 only as a placeholder for future findings (the box around all other variables in Figure 4), and we return below to a discussion of how the context could be investigated.

Taken together, Step 2 facilitates integration of previous theorizing by linking established theories to central moderators, and we have exemplified how and where this can be done (the exemplary links are depicted in Figure 4). Further theories can be linked to the model, depending on the specific outcome of interest. For example, to explain (dis)advantage framing effects on emotions, one could link the model to appraisal theories (Harth et al., 2008). This makes the model dynamic and open to further specification, depending on the specific research question. The model further helps specify how the cognitive focus evoked by (dis)advantage framing translates into specific responses, and which moderators may matter when. This concludes the outline of Steps 1 and 2 of our model, which is comprehensively summarized in Figure 4.

Below, we will describe the benefits of applying the two-step model in research practice. It can be used as an organizing framework for past and future work, to explain specific responses to (dis)advantage framing, as well as to develop novel predictions and a comprehensive research agenda.

### Applying the Two-Step Model to Specific (Dis)Advantage Framing Effects

A primary benefit of the two-step model is that it can be applied to any specific research question (and context), to predict effects of (dis)advantage framing on recipients’ reactions to inequality. This is achieved through the model’s links to moderators and established theories (see Step 2). This can help to resolve (seeming) contradictions in previous research, provide new interpretations of previous findings, and help work out predictions for novel research questions. We give examples of this below.

#### Resolving (Seeming) Contradictions

First, by considering the moderators of Step 2, the two-step model can reconcile inconsistent previous findings. To continue one example from above, SIT/SCT points at the importance of group membership as a moderator of (dis)advantage framing effects, and this may help to resolve some (seeming) contradictions. In particular, group membership can explain why disadvantage framing has sometimes led to less (Powell et al., 2005), and sometimes to more inequality acknowledgment (Bruckmüller et al., 2012). On the one hand, advantage framing is more self-relevant to advantaged groups and can threaten their higher status and identity—motivating them to downplay inequality more following advantage framing (Dover, 2022; Knowles et al., 2014). On the other hand, disadvantage framing is more self-relevant to disadvantaged groups and can threaten their self-esteem via internal attributions of worse outcomes (Lowery & Wout, 2010)—possibly motivating them to downplay inequality more following *dis*advantage framing. Applying the two steps of the proposed model may resolve this seeming contradiction. For the advantaged group, a cognitive focus on the ingroup is induced by advantage framing, and for the disadvantaged group, by disadvantage framing. The perceived size of inequality is then moderated by their group membership, with the cognitive focus on the ingroup leading to less inequality acknowledgment for both groups. Thus, the seeming contradiction that disadvantage *and* advantage framing can lead to less acknowledgment of inequality can be explained through the different cognitive foci these frames evoke (Step 1) and their interaction with the moderator group membership (Step 2), as the different cognitive foci translate into a focus on either the ingroup or the outgroup, depending on a recipient’s group membership, with predictable consequences for the perception of inequality.

Another contradictory finding from earlier research is that disadvantage framing can make inequality sometimes seem less legitimate (e.g., Dietze & Craig), but sometimes not (e.g., Braun et al., 2023b), and this can be resolved by considering size of inequality as a moderator. In particular, drawing on the combination of a cognitive focus on (dis)advantage and prospect theory explains why disadvantage framing only affects legitimacy when inequality is (perceived as) large (e.g., Bruckmüller et al., 2017a). Disadvantage framing evokes a cognitive focus on the negative side of inequality, and advantage framing on the positive side (Step 1). Since large losses are judged as more severe than large gains (but small losses and small gains are perceived to be similar in value), disadvantage framing makes only large (but not small) inequality seem less legitimate, compared to advantage framing (Step 2; cf. Kahneman & Tversky, 1979).

#### Providing New Interpretations for Previous Findings

Further, in combination with the predictions of cognitive focus in Step 1, each moderator-theory link in Step 2 can be applied to predict specific responses to (dis)advantage framing. In doing so, it also accommodates previous theoretical explanations of various effects and can in some cases suggest new interpretations for them. To illustrate, we will return once more to the examples that we used in the previous paragraph.

SCT suggests that advantage (disadvantage) framing facilitates self-categorization of advantaged (disadvantaged) people at the group level. One prediction that follows is that, for the advantaged, advantage framing induces more group-level emotions than disadvantage framing, and, indeed, Powell et al. (2005) found that it induces more collective guilt. Another prediction is that the disadvantaged will react more defensively to disadvantage than advantage framing because the former represents a stronger threat to their group-based self-esteem. This is indeed what Lowery and Wout (2010) found.

Another moderator-theory link in Step 2 was that between size of inequality and prospect theory. Large losses are perceived as more severe than large gains, while small losses and gains appear similar in value. From this follows the prediction that disadvantage framing of large inequality will be perceived as less legitimate than advantage framing of large inequality, and this is what Bruckmüller et al. (2017a) indeed found.

#### Making Novel Predictions

Most importantly, the moderator-theory links in Step 2 and their combination with Step 1 can also be used to make novel predictions. To illustrate this, we will give just one specific example of a hypothetical research project. Imagine that a researcher aims to address some of the research gaps identified in the systematic review. Specifically, she wants to investigate how disadvantaged group members react emotionally to (dis)advantage framing (because responses of members of disadvantaged groups have been studied much less than responses of members of advantaged groups), and she decides to focus on sexual orientation inequality (as this is an important inequality rarely studied in previous inequality framing research). To derive predictions, she starts by applying the first step of the model to her research question. The linguistic focus in her experiment lies on lesbian women (for her participants, the ingroup) in the disadvantage framing condition, and on straight women (the outgroup) in the advantage framing condition. According to the two-step model, this induces a matching cognitive focus in participants. Thus, our researcher expects the disadvantages of lesbian women to be more salient in the disadvantage framing condition, and the advantages of straight women to be more salient in the advantage framing condition.

This salience of different aspects of sexual orientation inequality then guides recipients’ responses in Step 2. Here, the researcher uses the model’s links to established theories to derive specific predictions. She is particularly interested in emotions, and appraisal theories of emotion posit that individuals base their emotional reaction to an issue, among other things, on the appraisal of whom the issue affects (Moors et al., 2013; see also Harth et al., 2008). She thus expects more ingroup-focused emotions in the disadvantage framing condition (e.g., more shame) and more emotions targeting straight women (the outgroup) in the advantage framing condition (e.g., more anger).

Finally, the two-step model points to the importance of the communicative context. The researcher identifies the political climate (i.e., widely shared attitudes and policies) toward queer and straight people in her country compared to others as a potential moderator (e.g., Bettinsoli et al., 2020). Thus, she decides to conduct the experiment in two countries that vary in the respective attitudes and policies (e.g., Germany and Italy, Borras Guevara et al., 2023). This allows her to test the generalizability of her hypotheses across contexts, or rather, to test the political climate as a moderator. Since previous studies have almost never systematically varied the context, and never the country, our hypothetical researcher thereby taps into an important research gap identified by the two-step model.

### Future Research Directions for the Study of (Dis)Advantage Framing Effects

Importantly, beyond the facilitation of predictions of specific (dis)advantage framing effects in specific context as illustrated in the previous paragraphs, the two-step model also points at several important next steps in inequality framing research more generally, of which we outline some below.

#### Test Step 1 Experimentally

A prime task for future research is to experimentally test Step 1, that is, whether a linguistic focus evokes a cognitive focus on either advantage or disadvantage, and whether it always does so or under which conditions it may not. Even though previous research that has used open responses as dependent variables has documented that participants are more likely to refer to the group the linguistic focus is on in their subsequent responses (Bruckmüller & Braun, 2020; Powell et al., 2005), there are some evident problems with inferring cognitive focus from participants’ verbal responses. One is that this confounds cognitive and linguistic focus. Presenting a disadvantage frame may make participants focus more on disadvantage in their response. However, since they express this focus through language, it is impossible to distinguish between cognitive and linguistic focus in their verbal response. Another problem is that verbal behavior can be seen as a *reaction* to (and not an expression of) different linguistic foci (see results of the systematic review). Relatedly, from the verbal response alone, we cannot differentiate between the cognitive process assumed in Step 1, and a more communicative explanation (cf. Grice, 1991). In particular, it is possible that participants, in response to disadvantage framing, focus more on this aspect because they assume that this is the relevant aspect which the communicator (or the experimenter) wants them to speak about (cf. Sher & McKenzie, 2006). A more direct test of cognitive focus could use reaction times, implicit associations, or eye-tracking to measure variables such as attention to or cognitive availability of salient aspects of inequality.

#### Systematically Investigate the Interplay of Variables in Step 2

Step 2 cannot and does not aim to predict the effects of (dis)advantage framing on a general level. Thus, to predict specific responses, the two-step model needs to be specified in two ways. First, one needs to consider which specific (further) moderators matter in a particular context (e.g., for particular outcomes, participants, location, and time). Second, mediators need to be investigated more rigorously, to understand the underlying mechanisms between cognitive focus and specific responses. Here, the two-step model facilitates the identification of systematic research gaps and promising candidates for future investigation, through its systematic categorization of such moderators and mediators. To illustrate this, Figure 4 leaves placeholders for further moderators and for mediators, and we explain below where the search for them may start.

##### Investigate Further Moderators

Situational moderators can be determined by looking more closely at variations in the framing manipulations. Beyond size, legitimacy, and level of inequality (which are already included in the two-step model), one likely candidate is inequality domain. Different domains (e.g., gender, class) differ from each other, for example, in perceived legitimacy and size. These aspects can moderate how people react to framing. Because of this, domain itself is likely also a moderator (see systematic review). Another candidate is the outcomes for which an inequality is described. Earlier studies often described inequality in positive outcomes (e.g., money) and less often in negative ones (e.g., financial problems). However, gains (positive outcomes) are perceived as less severe than losses (negative outcomes; Steiger & Kühberger, 2018), which could mean that inequality in positive outcomes is also perceived as less severe.

A starting point for further inter-individual moderators is pre-held attitudes. Some (isolated) studies suggest that attitudes moderate framing effects, but these findings were not (yet) consistent enough to include them in the two-step model. For example, political orientation moderated the support of redistributive taxes (Chow & Galak, 2012), egalitarianism moderated intentions to reduce inequality (Bruckmüller et al., 2017b), and racism moderated perceived threat (Puryear et al., 2019). A task for future research is to replicate and extend such findings.

##### Identify and Investigate Mediators

The two-step model also helps to identify mediators of (dis)advantage framing effects that could be systematically investigated in future research. Moreover, it highlights the need to clarify the status of central variables (such as size and legitimacy of inequality) as dependent variables, mediators, or moderators (or which status they have for which kinds of inequality, communicative contexts, and effects). This would help craft a more comprehensive understanding of (dis)advantage framing effects, and how they relate to each other (in sequence). In earlier studies, mediators were often only investigated once (for a particular dependent variable) and were never experimentally manipulated. Thus, no specific mediator could be included in the current version of our two-step model. Nevertheless, some mediators appeared repeatedly in the literature and warrant a closer look.

Based on the results of the systematic review, it seems plausible that some of the investigated dependent variables mediate the effect of framing on more distal variables. For example, disadvantage framing arguably shifts the cognitive focus to the disadvantaged group and may thus lead to more emotions targeted at the disadvantaged, such as sympathy, while advantage framing shifts the focus to the advantaged, and leads to emotions targeting them, such as guilt or pride, depending on contextual moderators such as the legitimacy of inequality (Harth et al., 2007). These emotions may then motivate more support for interventions (Braun et al., 2023b). Another example is that shifting the focus to disadvantages may also make inequality seem larger (e.g., Brown & Craig, 2020) and more unfair, depending on the perceived size of inequality (e.g., Bruckmüller et al., 2017a), and this may in turn lead to greater support for collective action (e.g., Dietze & Craig, 2021). Thus, shifting the focus to (dis)advantage may first influence rather proximal variables (e.g., emotions, perceptions of inequality), and these may, in turn, influence more distal responses (e.g., behavior[al intentions]), depending on moderators (e.g., size, legitimacy).

The following mediators, identified in isolated studies, appear to be promising starting points for a more systematic investigation. Negative affect mediated between disadvantage framing and higher perceived discrimination (Brown & Craig, 2020) and more modern racism (Powell et al., 2005). Other (dis)advantage framing effects were mediated by ingroup identification (e.g., Lowery et al., 2012) and features of inequality, such as size (Schnepf et al., 2023) and legitimacy (e.g., Dietze & Craig, 2021). Replicating and extending these mediators (also experimentally) would allow a more fine-grained classification into proximal (mediating) and more distal variables, and their integration into the two-step model.

#### Investigate the Communicative Context

Finally, (dis)advantage framing effects are embedded in their communicative context (the box around all other variables in Figure 4). To recap, we suspected that its features, such as the (country-level) size and legitimacy of inequality (cf. Bruckmüller et al., 2017a; Harth et al, 2008), or linguistic norms could influence framing effects (Dover, 2022). However, the context has never been explicitly investigated as a moderator, and we left a placeholder for it in Figure 4. It is an important task for future research to fill this placeholder with empirical evidence. The broader framing literature may provide starting points for specific features of the context which could be varied. For example, prior knowledge can moderate the effectiveness of emphasis frames (e.g., Jin & Han, 2014). In contexts where a certain inequality is rarely talked about in the media or in political debates, participants may have weaker pre-held attitudes toward it, and this may lead them to be more susceptible to framing effects. Another example is that cultural norms, such as the level of uncertainty avoidance in medical decision-making, can influence the effectiveness of gain/loss framing (e.g., in Middle-Eastern vs. Western contexts; Tabesh et al., 2019). Similarly, inequality-related norms, such as meritocracy beliefs vary between contexts (e.g., Mijs, 2021) and could moderate (dis)advantage framing effects. For example, privileged participants with a high belief in meritocracy could find advantage framing, and the associated focus on illegitimate ingroup privilege, especially uncomfortable (Chow et al., 2008). Thus, future studies should vary, or at least measure, (features of) the communicative context, and compare the effects of (dis)advantage framing across different normative contexts, in different languages, and for different inequalities. Importantly, this would also address a central research gap identified in the systematic literature review, namely the overwhelming focus on the United States and Western Europe.

### Contributions of the Two-Step Model in a Nutshell

In the previous sections, we have comprehensively developed and described the two-step model of (dis)advantage framing effects and have illustrated its benefits for research practice with specific examples. Below, we summarize the contributions.

First, the two-step model provides an explicit definition of (dis)advantage framing, grounded in framing theory (via the inclusion of linguistic focus). This definition may help to streamline manipulations of (dis)advantage framing, such that unsystematic variation in factors that may interact with framing effects can be prevented. In other words, the definition may help researchers to indeed only manipulate whether inequality is presented as disadvantage or as advantage, without unintentionally varying other persuasive features of their message (Scheufele & Iyengar, 2014). Second, the two-step model distinguishes between the linguistic focus in descriptions of inequality and the cognitive focus this evokes in recipients (Step 1), and in doing so, provides an explicit explanation of *why* (dis)advantage framing effects occur. Third, the model categorizes subsequent responses and moderators, and fourth, it connects them to established theories (Step 2). It can thereby help to predict specific effects in specific contexts, account for inconsistent findings of previous studies, and help identify important next steps for future research. Fifth, as a result of this, the model can act as a blueprint for future studies, as it illustrates what potential moderators to watch out for in future research and when. It can also help making more systematic predictions in future research and integrating them across domains, outcomes, and other aspects of inequality that have varied rather unsystematically in previous research. Finally, the two-step model is not only designed to systematize the complex landscape of (dis)advantage framing research, but it is also conceived as a dynamic framework intended to evolve, expand, and be refined through future empirical and theoretical advancements.

In conclusion, the empirical evidence gathered so far does not allow *general* predictions of the “processing and reactions,” which would follow a cognitive focus on disadvantage or advantage. Instead, Step 1 makes a general prediction of why (dis)advantage framing effects occur, and Step 2 can be specified to derive highly context-dependent predictions for (dis)advantage framing effects for a particular kind of inequality, communicated in a particular context, and on particular variables of interest.

## General Discussion

To help integrate and advance the emerging field of disadvantage framing research, we conducted a systematic literature review of (dis)advantage framing effects and developed the two-step model to integrate these effects on a conceptual level. The review identified unsystematic methodological variations in the design of previous studies that may have biased framing effects and led to (seemingly) inconsistent results. We have also described relatively consistent (dis)advantage framing effects and (hidden) moderators. The two-step model explains why (dis)advantage framing affects recipients’ responses, and it systematizes central moderators, mediators, and dependent variables, which helps to make specific predictions on (dis)advantage framing effects in specific communicative contexts, to explain inconsistent earlier findings, and to point out directions for future research. Below, we discuss the implications and limitations of the review and the two-step model together.

### Practical Implications for Practitioners and Researchers Addressing Inequality

In the Introduction, we asked how problematic a dominant disadvantage framing really is and which effects it may have to use more advantage frames. Based on the results of the systematic review, we conclude that neither disadvantage nor advantage framing are per se more conducive to challenging or maintaining inequality. For example, disadvantage framing can make inequality seem less legitimate (Dietze & Craig, 2021), but can also lead to more stereotypical evaluations of disadvantaged groups (Bruckmüller et al., 2012). Advantage framing can increase collective guilt and lower modern racism (Powell et al., 2005), but can also lead to reactance among the advantaged (Phillips & Lowery, 2015). Further, the two-step model explains why such a general implication of (dis)advantage framing is an unrealistic expectation, as any effect beyond salience (i.e., cognitive focus) likely depends on the specific context and associated moderators. This realization has important implications for practitioners and researchers addressing inequality.

#### For Practitioners Addressing Inequality

One implication of the systematic review and of the two-step model is that addressing inequality one-sidedly, that is, either only from a perspective of disadvantage or only of advantage, can have unintentional, potentially undesirable effects. This is critical because most previous research on inequality and many interventions have focused on disadvantage and disadvantaging mechanisms to understand and to challenge inequality to the neglect of advantage and advantaging mechanisms (Phillips & Jun, 2022). Although less frequent, efforts to increase privilege awareness are also growing, for example, in the form of privilege checklists and video interventions (Case et al., 2014; Ehrke et al., 2020).

Based on the results of the systematic review and theoretical consideration inspired by the two-step model, we would expect interventions to be most effective if they simultaneously address disadvantage *and* advantage. For example, confronting people (also) with advantage may buffer against the negative effects of disadvantage framing. Disadvantage framing makes people focus on the disadvantaged to explain inequality (Bruckmüller & Braun, 2020), which may lead to more (availability of) stereotypes against disadvantaged groups (Bruckmüller et al., 2012). Simultaneous confrontation with advantage framing may lead people to focus (also) on advantaged people, which may reduce one-sided, stereotyped views.

In turn, disadvantage framing may buffer against the negative effects of advantage framing. For example, considering the intersectionality of one’s privileged *and* disadvantaged identities may prevent people from reacting defensively to privilege confrontations (cf. Phillips & Lowery, 2015). Indeed, some privilege awareness exercises prompt participants to identify both aspects of their identities and then reflect which consequences these have on their lives. Other interventions let participants simulate the experience of privilege and oppression in the classroom. For example, Ehrke et al. (2020) gave some participants an advantage and others a disadvantage based on their seat in the classroom in a ball-throwing competition. Pickering (2023) let participants compete to build a tower and (dis)advantaged some groups by giving them less or more materials. Based on this experience, the participants then reflected on their own privileged and disadvantaged identities in their real lives. These interventions increased privilege awareness, which in turn improved outgroup attitudes (Ehrke et al., 2020), enabled participants to analyze systems of inequality and their own position in it (qualitative analysis; Rabelo et al., 2023), and motivated them to take action against inequality (qualitative analysis; Pickering, 2023). An important caveat is that these studies used either no control group (Pickering, 2023; Rabelo et al., 2023), or one where participants did not engage with inequality at all (Ehrke et al., 2020).

In sum, we would expect such interventions, that is, those which focus on both disadvantage and advantage, to be most effective in challenging inequality. Future research should empirically compare the effectiveness of separate disadvantage and advantage confrontation, compared to their combination.

#### For Researchers Addressing Inequality

The present findings also have important implications for researchers addressing inequality. As mentioned above, there is a chronic disadvantage lens in existing scholarship on inequality that does not accurately reflect the mechanisms creating and maintaining inequality (Phillips et al., 2022) and which may obscure possible solutions (Nixon, 2019). The following example illustrates how this can obscure advantaging mechanisms as explanations of inequality and as starting points for interventions. Major et al. (1998) found that information about bias against minority groups caused Black students to disengage their self-esteem from intellectual feedback, while White students remained engaged. The authors concluded that White students would be relatively unaffected by information about racial bias because they would not experience it as much (Major et al., 1998). However, Lowery and Wout (2010) later showed that evidence of racial inequality *need not* cause disadvantaged groups to disengage, and that it *can* also cause advantaged groups to disengage – namely, when inequality was framed as advantage. This may be the case because advantage framing made salient how the outcomes of advantaged groups are also tied to bias (in terms of favoring). These findings helped dispute (stereotypical) claims about disadvantaged groups, such as that women or racial minorities respectively devalue mathematics or academic achievements more generally. Thus, efforts to increase the representation of disadvantaged groups by increasing the value they place in these domains may be misguided (Lowery & Wout, 2010), and interventions may be more effective if they also target the overrepresentation of advantaged groups.

In sum, considering advantaging mechanisms can render often overlooked mechanisms of inequality more visible, prevent false conclusions, and allow constructing more effective interventions against inequality. It is thus crucial to know whether and to what extent (inadvertently) conceptualizing inequality as (dis)advantage can affect how participants understand and react to inequality and how it can thus guide (and bias) inequality research. The present work provides insights into this by providing an overview of consistent (dis)advantage framing effects and (hidden) moderators as well as a framework that helps identify further candidates for previously overlooked moderators and mediators in a more systematic way.

### Limitations and Future Research Directions

In the systematic literature review, we were specifically interested in effects of *equivalent* advantage versus disadvantage frames, so we did not include studies exclusively comparing disadvantage *or* advantage frames to a control group (no focus frames, i.e., descriptions of inequality that focus on no particular group). However, no focus frames are also a prevalent frame for some inequalities (e.g., wealth inequality, “there is a wealth gap between the poor and the rich”; Jun et al., 2022) and should be reviewed in future work. We also would not have included studies on simultaneously presented (competing) frames. Few studies have investigated the use of competing frames at all (Borah, 2011; Chong & Druckman, 2007b), and only one study that we know of speaks at least somewhat to the effect of competing equivalent inequality frames (Braun et al., 2023a). Research on competing frames would, at least for some kinds of inequality, more closely mirror (dis)advantage framing in public discourse because recipients are often simultaneously exposed to different frames of issues, especially in competitive communication, such as journalism or politics (Chong & Druckman, 2007a) and different inequality domains vary in the extent to which one particular framing dominates public discourse (Jun et al., 2022).

Further, the two-step model cannot make general predictions of (dis)advantage framing effects. Rather, the second step needs to be specified for specific contexts and dependent variables. While this is a limitation to some extent, we would argue that the complexity of framing effects in and of itself defies such general predictions and that the model reflects this reality. Moreover, this openness to further specification may also render the model a “blueprint” for equivalency framing effects more generally, that is, beyond the context of inequality and its framing. Consider gain-loss framing as an example. In the classical “disease problem,” participants prefer a safe public health program when outcomes are framed positively (e.g., lives saved), but prefer a risky program when framed negatively (e.g., lives lost). One explanation for this is that gain frames invoke positive emotions, and loss frames negative emotions, and that this emotional response guides subsequent reactions (Nabi et al., 2020). Applying our two-step model, the primary emotional response can be understood as an emotional *focus*, evoked by a linguistic focus, on either gains or losses (Step 1). How exactly people react to gain-loss frames then depends on a range of moderators, which parallel the categories of the two-step model (Step 2). Research has identified situational (e.g., features of the risk; Steiger & Kühberger, 2018) and inter-individual moderators of gain-loss framing, including features of the recipient (e.g., motivation; Godinho et al., 2016) and features of the communicators (e.g., an authority vs. laypeople; Yang et al., 2024). The moderators included in the two-step model can inform the search for further moderators of gain-loss framing and vice versa. Thus, a valuable next step would be to generalize the two-step model into a comprehensive framework integrating various equivalency framing paradigms.

Finally, future work should extend the proposed theoretical model with theorizing on when and why people use advantage or disadvantage frames (i.e., the path from inequality to linguistic focus in Figure 4). This question is beyond the scope of the current work, which systematizes and integrates previous research on *effects* of (dis)advantage framing, so we only mention two prominent approaches here. One is based on group interests and suggests that disadvantage frames are preferred by both advantaged groups (because it shifts threatening attention away from their privilege; see Dover, 2022) and disadvantaged groups (because it draws attention to their plight and may raise sympathy; see Harth et al., 2008). The other is based on norm theory and posits that people are more likely to compare non-normative, lower-status groups to normative, higher-status groups than vice versa, which translates to disadvantage framing (Hegarty & Bruckmüller, 2013; Hegarty & Pratto, 2001).

### Citations Statement

Our search strategy was based on a list of English keywords that may not have been exhaustive and was presumably influenced by our subjective prior knowledge of relevant studies and the language used therein. The search strategy may thus have missed studies from different research fields or methodological approaches using other language conventions. We tried to counteract this by compiling a very broad set of keywords (see Supplemental Table B1), combining references to inequality and framing. This returned a vast corpus of mostly non-relevant manuscripts for screening, but reduced the odds of missing relevant studies. It was also necessary because many articles on (dis)advantage framing did not mention framing as keyword or in their abstract. The authors of included studies were based mostly in the United States and Western Europe, which is problematic because framing effects may depend on the sociocultural context.

### Constraints on Generality Statement

The findings of the systematic literature review are restricted to (mostly) European and U.S. samples, and specific Germanic and Romance languages. Further, the identified moderators illustrate that there is not *the* general (dis)advantage framing effect. Instead, specific responses to (dis)advantage framing depend on features of the inequality, the communicators, and the recipients in question. The two-step model (in its current form) can, therefore, only be applied to recipients’ reactions to (dis)advantage frames of social inequality, and only to languages which allow (dis)advantage framing, even though future work may develop it further to encompass other kinds of framing effects (see above).

## Conclusion

In public discourse and research on inequality, there is a pervasive and one-sided focus on disadvantage. Therefore, it is crucial to understand whether it matters if we construe inequality in terms of advantages or disadvantages. The present research illustrates that (dis)advantage framing critically shapes how recipients perceive and react to the issue, even if we do not fully understand yet when and how exactly this happens in which contexts. The proposed two-step model advances our understanding of why (dis)advantage framing effects occur and systematizes central responses and moderators. It aims to integrate previous theorizing and to make studies more comparable on a methodological and conceptual level, ultimately creating a more comprehensive understanding of the complex issue of inequality and its framing.

## Notes

1. In the categories Psychology, Economics, Linguistics, Sociology, Social Issues, Social Sciences Interdisciplinary, Business.

2. Note that, in (dis)advantage framing research, there is no unified terminology and central terms sometimes overlap with other research fields (e.g., the term “frame” also means a “digital data transmission unit” in telecommunication, and one of many “still images,” which produce a moving picture). Thus, in order to capture any relevant research, it was necessary to use a very broad set of keywords. This resulted in a vast corpus of mostly irrelevant manuscripts (see below) which did, however, include all relevant papers that we were already familiar with.

3. Supplemental Tables B6–B18 comprehensively report all included effects. These tables display summary statistics in the focal experimental groups (advantage vs. disadvantage framing). They also report the direction, effect sizes, and confidence intervals of all framing main effects, as well as all investigated moderators and mediators (and whether they were significant). The findings in these tables are marked as reported or calculated by us from available data, as pre-registered (or not), and as hypothesized versus exploratory.

4. Differences in publishing rates between significant and non-significant effects appeared to be small. Significant framing main effects were more often included in peer-reviewed, published manuscripts (i.e., in journals, books, or conference papers, 57.0%) than in non-peer-reviewed or unpublished ones (i.e., online repositories, theses, unpublished data, 43.0%), as were significant interaction effects (53.3% vs. 46.7%). The reverse was true for non-significant main effects, which were more often included in non-peer-reviewed or unpublished manuscript (51.6%) than in peer-reviewed ones (48.4%). However, non-significant interaction effects were more often published with peer-review (55.7%) than not (44.3%).

5. Note that we categorized dependent variables on a more abstract level here than in the results section of the systematic review. This is because specific sub-categories, such as ingroup identification and group evaluations, were researched relatively often, warranting reporting them separately there.

6. Note, however, that the focus in this study was on general gender differences (e.g., in leadership style; preferred activities to relax); inequality only featured as a side note, and more so in the leadership than in the leisure condition (e.g., male managers being evaluated less critically). This shows, again, how stark a research gap the communicative context is.
